# A new species of *Heremites* Gray, 1845 (Squamata, Scincidae) from the transboundary Hindu Kush foothills

**DOI:** 10.3897/zookeys.1291.1863652

**Published:** 2026-09-04

**Authors:** Daniel Jablonski, Sylvia Hofmann, Muhammad Idrees, Muazzam Ali Khan, Qaisar Jamal, Wolfgang Böhme, Rafaqat Masroor

**Affiliations:** 1 Department of Zoology, Comenius University in Bratislava, Ilkovičova 6, 84215 Bratislava, Slovakia Department of Zoology, Comenius University in Bratislava Bratislava Slovakia; 2 Herpetological Collection, 1st Zoological Department, Natural History Museum Vienna, Burgring 7, 1010 Vienna, Austria Herpetological Collection, 1st Zoological Department, Natural History Museum Vienna Vienna Austria; 3 Leibniz Institute for the Analysis of Biodiversity Change, Museum Koenig, Adenauerallee 127, 53113 Bonn, Germany Leibniz Institute for the Analysis of Biodiversity Change, Museum Koenig Bonn Germany; 4 Institute of Zoological Sciences, University of Peshawar, 25120 Peshawar, Khyber Pakhtunkhwa, Pakistan Institute of Zoological Sciences, University of Peshawar Peshawar Pakistan; 5 Department of Zoology, University of Gujrat, Hafiz Hayat Campus, Jalalpur Road, Gujrat, Pakistan Department of Zoology, University of Gujrat Gujrat Pakistan; 6 Zoological Sciences Division, Pakistan Museum of Natural History, Shakarparian, Islamabad, Pakistan Zoological Sciences Division, Pakistan Museum of Natural History Islamabad Pakistan

**Keywords:** Afghanistan, biogeography, *
Mabuya
*, Pakistan, Palearctic, taxonomy, *
Trachylepis
*

## Abstract

The Western Palearctic genus *Heremites* Gray, 1845 (Scincidae) has a long history of taxonomic uncertainty, with particularly unstable placement of the disjunct Hindu Kush populations reported from eastern Afghanistan and northwestern Pakistan. Herein this eastern population is reassessed using an integrative dataset combining broad mitochondrial screening, a six-locus phylogeny, and comparative morphology. Phylogenetic analyses recovered four main well-supported clades corresponding to *H. auratus*, the *H. septemtaeniatus* complex, the *H. vittatus* complex, and the Hindu Kush lineage. The Hindu Kush lineage is inferred to be deeply divergent and was recovered as sister to the clade comprising *H. auratus* and the *H. septemtaeniatus* complex, with an estimated split in the Late Miocene at approximately 10 Mya. This deep mitochondrial divergence is further supported by concordant structuring in the nuclear allele networks. Uncorrected p-distances further support its distinctiveness, with high mitochondrial divergence from western clades (15.2–15.8% in cyt*b*; 17.0–21.0% in ND2) and comparatively lower but consistent nuclear differentiation (1.17–2.59% in MC1R and 2.5–3.3% in RAG1). Multivariate analyses of morphometric, meristic, and categorical data separate the Hindu Kush lineage from *H. auratus* and the *H. septemtaeniatus* complex, supporting the genetic data. We therefore describe this eastern lineage as a new species, currently known from two foothill localities along the southern margin of the Hindu Kush, and discuss its biogeographic significance and the need for a further genus-wide integrative revision.

## Introduction

The Western Palearctic harbours reptile genera whose ranges appear continuous at broad scale but, upon closer inspection and application of DNA data, comprise divergent populations separated by major biogeographic barriers (Sindaco and Jeremchenko 2008; Khan et al. 2021; Pola et al. 2024; Burriel-Carranza et al. 2025). Such patterns seem especially frequent along the Palearctic–Oriental transition, where topographic complexity, climatic gradients, and historical biogeographic connectivity have repeatedly promoted persistence in old refugia following lineage divergence or historical colonisations (Agarwal et al. 2022; Masroor et al. 2022; Hofmann et al. 2023; Jablonski et al. 2025). The Hindu Kush and adjacent orographic units appear to constitute a particularly strong biogeographic filter, possibly reflecting past climatic conditions and resulting in “outlier” occurrences whose taxonomic identity and evolutionary origins often remain uncertain in the absence of dedicated integrative fieldwork and genetic data (e.g. Jablonski et al. 2021, 2026; Lee et al. 2023).

This pattern may also apply to the genus *Heremites* Gray, 1845 (Scincidae). This genus is currently treated as a Western Palearctic clade of the family Scincidae with three recognised species, *H. auratus* (Linnaeus, 1758), *H. septemtaeniatus* (Reuss, 1834) and *H. vittatus* (Olivier, 1804) distributed from North Africa through the Middle East and Anatolia to Central Asia (Sindaco and Jeremchenko 2008; Šmíd et al. 2014; Candan et al. 2019). However, despite the apparent breadth of the range, the distribution is not fully continuous, and the taxonomy remains challenging (Akhmedov and Szczerbak 1987; Faizi and Rastegar-Pouyani 2006; Güçlü et al. 2014; Bahmani et al. 2016, 2018) because of morphological similarity among species (and possible admixture) and ongoing uncertainty about the status and limits of named forms across the eastern part of the range (Anderson 1999). These difficulties have resulted in inconsistent treatments of key taxa and emphasise the need for renewed DNA-based evaluation.

At the eastern edge of the genus distribution, *H. septemtaeniatus* has long been central to taxonomic ambiguity and was historically considered a subspecies of morphologically similar *H. auratus* from the central and western part of the genus range (see discussion by Masroor et al. 2021). A single available Afghan specimen (ZFMK 9064; the second one ZMK 2572 was destroyed) from Nangarhar Province has been highlighted as a biogeographic enigma (Moravec et al. 2006), being separated from the core range of the genus by the Hindu Kush and intervening desert basins, with large geographic gaps (~700 km) to the nearest expected or reported occurrences in northwestern Afghanistan, central Iran or southern Turkmenistan (Sindaco and Jeremchenko 2008; Šmíd et al. 2014; Wagner et al. 2016; Masroor et al. 2021). In addition to the spatial disjunction, the taxonomic placement of eastern populations has been unstable, including conflicting assignments of the name *transcaucasicus* (Chernov 1926) and debate over its relationship to *H. septemtaeniatus* vs *H. auratus* (Akhmedov and Szczerbak 1987; Moravec et al. 2006). Against this backdrop, the discovery of *Heremites* in Afghanistan (Moravec et al. 2006) and northwestern Pakistan (Masroor et al. 2021) provided a critical new piece of evidence for the eastern limits of the genus and the structure of variation in the *H. septemtaeniatus* complex that should be better investigated. However, the available material has been limited.

Masroor et al. (2021) reported a reproducing population from Lower Dir District, Khyber Pakhtunkhwa Province, Pakistan (Shah Alam Baba area; ca 1,110 m a.s.l.), based on a series of specimens collected between 2013 and 2019. This record extended the known range eastward from the Afghan locality by ~140 km and placed *Heremites* in the foothills of the Hindu Kush near the Palearctic–Oriental transition zone (Sindaco and Jeremchenko 2008). It reinforces the region as a contact area where Western Palearctic reptile evolutionary lineages approach (and sometimes overlap with) Oriental assemblages (see Jablonski et al. 2024). In this context, the long-term oversight of *Heremites* in the region may partly reflect regional co-occurrence with the ecomorphologically similar Oriental species *Eutropis trivittata* (Hardwicke & Gray, 1827), increasing the likelihood of misidentification. Morphological assessment indicated close similarity between Pakistani specimens and the single Afghan voucher, with only minor differences in a small subset of characters, leading Masroor et al. (2021) to provisionally assign the Pakistani population to *H. septemtaeniatus transcaucasicus* (Chernov, 1926). Importantly, the same study also noted that some traits observed in the Pakistani–Afghan material deviate from what is typically characterised for the nominate *H. septemtaeniatus* and/or *transcaucasicus*, and explicitly argued that the disjunct distribution coupled with morphological peculiarities warrants further research, including DNA data, to test whether the isolated Hindu Kush population represents a relict or an unknown taxon.

These observations establish a clear taxonomic and biogeographic gap. First, the easternmost *Heremites* populations remain represented by limited comparative material from Afghanistan and by a geographically narrow sample from Pakistan, while much of the intervening region is unexplored or lacks vouchered records. Second, the provisional subspecific placement was necessarily conservative in the absence of molecular evidence and dense sampling across the complex. Third, the Palearctic–Oriental margin setting and the strong barrier effects of the Hindu Kush make the eastern lineage a plausible candidate for long-term isolation and independent evolutionary history (e.g. Agarwal et al. 2014, 2022), with direct implications for reconstructing range dynamics of Western Palearctic squamates, particularly Scincidae diversification (Karin et al. 2016).

In this study, we thus re-evaluate the Pakistani population of *Heremites* using DNA data to resolve its taxonomy. Building on the morphological baseline and biogeographic hypotheses proposed by Masroor et al. (2021), we (i) incorporate molecular evidence to assess its phylogenetic distinctiveness relative to named congeners, (ii) assess diagnostic morphological variation of the Pakistani lineage in a comparative context, and (iii) provide a formal taxonomic treatment.

## Materials and methods

### DNA sequence data

We selected six loci for subsequent phylogenetic analyses: 12S rRNA (12Sa-L, 12Sb-H; Kocher et al. 1989), 16S rRNA (16Sbr-L, 16Sbr-H; Palumbi 1991), cytochrome *b* (cyt*b*; MAB1, MAB2; Miralles and Carranza 2010), NADH dehydrogenase subunit 2 (ND2; MetF1, COI1R1; Arévalo et al. 1994; Macey et al. 1997), melanocortin receptor 1 (MC1R; MC1R.F, MC1R.R; Pinho et al. 2010) and recombination-activating gene 1 (RAG1; RAG1skinkF2, RAG1skinkF370, RAG1skinkR2, RAG1skinkR1200; Portik et al. 2010). Laboratory procedures generally followed Miralles and Carranza (2010), Güçlü et al. (2014), and Karin et al. (2016). Total genomic DNA was extracted using the E.Z.N.A.® Tissue DNA Kit following the manufacturer’s protocol. Newly generated sequences are deposited in GenBank (Suppl. material 1: tables S1–S3).

To place the Hindu Kush population within the phylogenetic framework of *Heremites*, we employed a two-stage analytical strategy. First, we assembled an extensive cyt*b* dataset comprising 209 terminals: 195 *Heremites* sequences listed in Suppl. material 1: table SS1, including 13 newly generated sequences, and 14 outgroup sequences. Cytochrome *b* was selected for the initial screening because it provides the densest available sampling of *Heremites* in terms of both geographic coverage and number of individuals (Carranza and Arnold 2003; Güçlü et al. 2014; Baier et al. 2017; Bahmani et al. 2018; Suppl. material 1: fig. S1).

Second, based on the cyt*b* screening, we assembled a multi-locus matrix by combining a curated subset of available GenBank sequences for the focal loci with our newly generated data, to assess the phylogenetic placement of the Hindu Kush population using both mitochondrial and nuclear markers. The final matrix comprised 57 terminals represented by 238 locus sequences, of which 169 were obtained from previous studies, and 69 were newly generated across the six target loci (Suppl. material 1: table SS2). Within *Heremites*, the dataset (published and newly generated) included one sample of *H. auratus*, four samples representing the *H. septemtaeniatus* complex, 13 representing the *H. vittatus* complex, and one sample representing the newly described Hindu Kush lineage from Pakistan.

### Phylogenetic analyses

We mapped the distribution of currently recognised *Heremites* species based on cyt*b* genotyping. All available *Heremites* cyt*b* sequences, together with representative outgroups, were compiled and imported into SeaView 5 (Gouy et al. 2021) for alignment construction and manual curation. The alignment was inspected and edited by eye to maintain reading-frame consistency and to minimise obvious misalignments. Because cyt*b* fragments did not fully overlap among studies, particularly those from Baier et al. (2017), sequence termini were harmonised by trimming and fitting to the reference sequence EU443142 (*H. vittatus*), resulting in a final alignment of 1,154 bp. Any remaining terminal regions with inconsistent overlap were coded as missing data (gaps/ambiguities).

This cyt*b* screening dataset was used to delineate major mitochondrial clades across the genus and to define the placement of the Hindu Kush lineage relative to published diversity. Given that *Heremites* taxonomy remains unresolved and mitochondrial structuring appears deep in parts of the genus, especially within *H. septemtaeniatus* and *H. vittatus*, we treated the principal lineages reported by Baier et al. (2017) and Bahmani et al. (2018) as operational units and refer to them as the *H. septemtaeniatus* and *H. vittatus* species complexes. Because the inclusion of short cyt*b* fragments (from Baier et al. 2017) affected overall support values and, in some trial runs, altered relationships, we additionally evaluated analyses with these short sequences included and excluded. Although the resulting topologies differed in some lower-level relationships and node support was reduced when short fragments were retained, species-level assignments of terminals remained consistent and were subsequently used for species-level mitochondrial lineage mapping.

Maximum-likelihood analyses were conducted in IQ-TREE v. 3.0.1 (Wong et al. 2026). The cyt*b* alignment was partitioned by codon position (1^st^, 2^nd^, 3^rd^), and the best-fit substitution models and, where appropriate, a merged partitioning scheme were selected using ModelFinder (Kalyaanamoorthy et al. 2017) under BIC with MFP+MERGE. Tree inference used IQ-TREE’s standard heuristic search, including iterative optimisation of candidate trees and re-estimation of model parameters. Node support was evaluated using Shimodaira–Hasegawa-like approximate likelihood ratio test (SH-aLRT) (Guindon et al. 2010) and ultrafast bootstrap (UFBoot; Minh et al. 2013; 1000 replicates each; --alrt 1000, -B 1000), and analyses were run with automatic thread selection (-T AUTO).

For the concatenated dataset, sequences of appropriate outgroup taxa were downloaded from GenBank, retaining only taxa for which sequences were available for at least three of the six target loci. An exception was made for *Chioninia vaillantii*, which was represented by two loci and retained because it was required for the corresponding secondary calibration. For several outgroup species, concatenated terminals were assembled from sequences obtained from different conspecific individuals because no single voucher was represented across the required loci (Suppl. material 1: table SS2). Newly generated sequences were combined with these data, and loci were concatenated following alignment. The final concatenated dataset comprised 57 taxa and had a total length of 4,814 bp (12S, 368 bp; 16S, 528 bp; cyt*b*, 1,146 bp; ND2, 943 bp; MC1R, 680 bp; RAG1, 1,149 bp).

Divergence times were estimated under a Bayesian tree framework in BEAST2 v. 2.7 (Bouckaert et al. 2019) using the concatenated dataset (4,814 bp; 57 taxa). We imposed the following five secondary time constraints from the fossil-calibrated phylogeny of mabuyine skinks by Pereira and Schrago (2017). All constraints were implemented through lognormally distributed priors: i) MRCA (Most Recent Common Ancestor) of *Ablepharus* and the core mabuyine radiation: 41.7 Mya (range 25.9–67.0; M = 3.73, σ = 0.24); ii) MRCA of *Chioninia delalandii* and *C. vaillantii*: 8.3 Mya (range 4.4–15.9; M = 2.12, σ = 0.33); iii) MRCA of *Eutropis longicaudata*, *E. multifasciata*, *E. rudis*, *E. rugifera*, *E. macularia*, *E. indeprensa*: 25.3 Mya (range 16.1–39.7; M = 3.23, σ = 0.23); iv) MRCA of *Heremites vittatus*, *H. septemtaeniatus*, *H. auratus*, and our newly identified *Heremites* lineage: 12.2 Mya (range 6.1–24.3; M = 2.5, σ = 0.35); v) MRCA of *Cryptoblepharus* and *Caledoniscincus*: 24.0 Mya (range 15.3–37.8; M = 3.18, σ = 0.231). We performed a BEAST2 run with a chain length of 50 million, a thinning interval of 5,000, a lognormal relaxed clock model, a birth-death tree prior, and a random starting tree. Substitution models were inferred using bModelTest (Bouckaert and Drummond 2017). Convergence and stationarity were verified with Tracer, with effective sample size (ESS) values > 200 for all traces. The final tree was obtained with TreeAnnotator v. 2.7 and visualised with FigTree v. 1.4.4 (Rambaut 2018).

Separate alignments of the protein-coding nuclear genes RAG1 (*n* = 15; 523 bp) and MC1R (*n* = 9; 599 bp) were analysed to assess evidence for independent allele differentiation (Suppl. material 1: table S3). Alleles were phased from heterozygous single-nucleotide polymorphisms using the PHASE algorithm (Stephens et al. 2001), as implemented in Hapsolutely v. 0.2.3 (Vences et al. 2024), with phase (-p) and allele (-q) probability thresholds of 0.5. Allele networks were reconstructed using the TCS algorithm (Templeton et al. 1992).

The *Heremites* sequences in the concatenated dataset were used to calculate uncorrected pairwise distances between lineages/species in MEGA X (Kumar et al. 2018), separately for the protein-coding markers cyt*b*, ND2, MC1R, and RAG1.

### Morphological data evaluation

To document and compare morphological differentiation, we measured 27 specimens of the genus *Heremites* curated at the Pakistan Museum of Natural History (**PMNH**), Pakistan, Natural History Museum in Vienna (**NHMW**), Austria and the Museum Koenig Bonn (**ZFMK**), Germany, representing both sexes, of known geographic origin, and assigned to particular species according to the distribution and species identification. Published data from two specimens of *H. vittatus* (Candan et al. 2019) were included only for descriptive comparison (Suppl. material 1: text). Thirty-seven (37) characters (23 morphometric, 10 meristic, and 4 qualitative) were measured with a digital calliper (0.1 mm precision) or evaluated, generally following Faizi and Rastegar-Pouyani (2006), Faizi et al. (2010), and Masroor et al. (2021): **SVL** (snout-vent length, from tip of snout to the anterior edge of cloaca); **TL** (tail length, from posterior edge of cloaca to the tip of the tail); **HL** (head length, distance between retroarticular process of jaw and snout-tip); **HW** (head width, measuring widest part of the head); **HH** (head height, from occiput to underside of jaws); **TrL** (trunk length, distance from axilla to groin measured from posterior edge of forelimb insertion to anterior edge of hindlimb insertion); **OD** (orbital diameter, the vertical diameter of orbit); **EL** (ear length, longest dimension of ear opening); **DN** (distance between nostrils); **END** (eye-nostril distance, distance between anterior corner of eye and tip of snout); **EED** (eye-ear distance, from posterior edge of eye to anterior corner of ear); **FrW** (frontal width); **FrL** (frontal length); **FnW** (frontonasal width); **FnL** (frontonasal length); **LorWa** (width of anterior loreal); **LorWp** (width of posterior loreal); **LL** (loreal length); **IpL** (length of interparietal); **MnW** (width of mental); **MnL** (length of mental); **HLL** (hindlimb length, length of femur and crus to tip of fourth toe); **FLL** (forelimb length, length of humerus and forearm to tip of fourth finger), **SL** (supralabials), **IL** (infralabials), **SSLE** (number of scales between last supralabial and ear opening), **PN** (pair of nuchals), **SAB** (scales across the body, number of scales in a single row around the widest part of the body), **DSNV** (dorsal scales in a row from first nuchal to above level of the vent), **VT** (ventral transverse, scales counted in a row from chin shields to cloaca), **SC** (number of subcaudals from behind vent to tip of tail), **EP** (ear pectination, number of scales projecting inside the ear opening), **KDS** (number of keels on dorsal scales), **SOF** (contact between the third supraocular and the frontal), **PFC** (prefrontals in contact or not), **PSC** (parietal shields in contact or not), and presence of white spots (**WS**).

All statistical analyses related to morphology were conducted using R 4.3.3 (R Core Team 2024). We included data from 25 specimens in the multivariate analyses: the newly discovered lineage (n = 14), *H. auratus* (n = 6), and the *H. septemtaeniatus* complex (n = 5). Analyses were conducted for a combined dataset of both sexes, because of the small sample size per group. Metric characters were size normalised using the allom function from the GroupStruct (Chan and Grismer 2021) package, applying normalisation at the species level (type = “species”). The resulting allometry-corrected dataset was subsequently used in downstream multivariate analyses. To jointly analyse heterogeneous character types (metric, meristic, and categorical), we performed Multiple Factor Analysis (MFA) using the FactoMineR package (Le et al. 2008); results were visualised with ggfortify (Tang et al. 2016) and ggplot2 v. 3.5.0 (Wickham 2016). To focus specifically on patterns in the metric characters, we conducted a Principal Component Analysis (PCA) on the normalised metric subset using prcomp with centring and scaling. Eigenvalues and explained variance were inspected using standard PCA summaries and eigenvalue extraction. PCA results were visualised using ggplot2.

## Results

**Figure 1.**
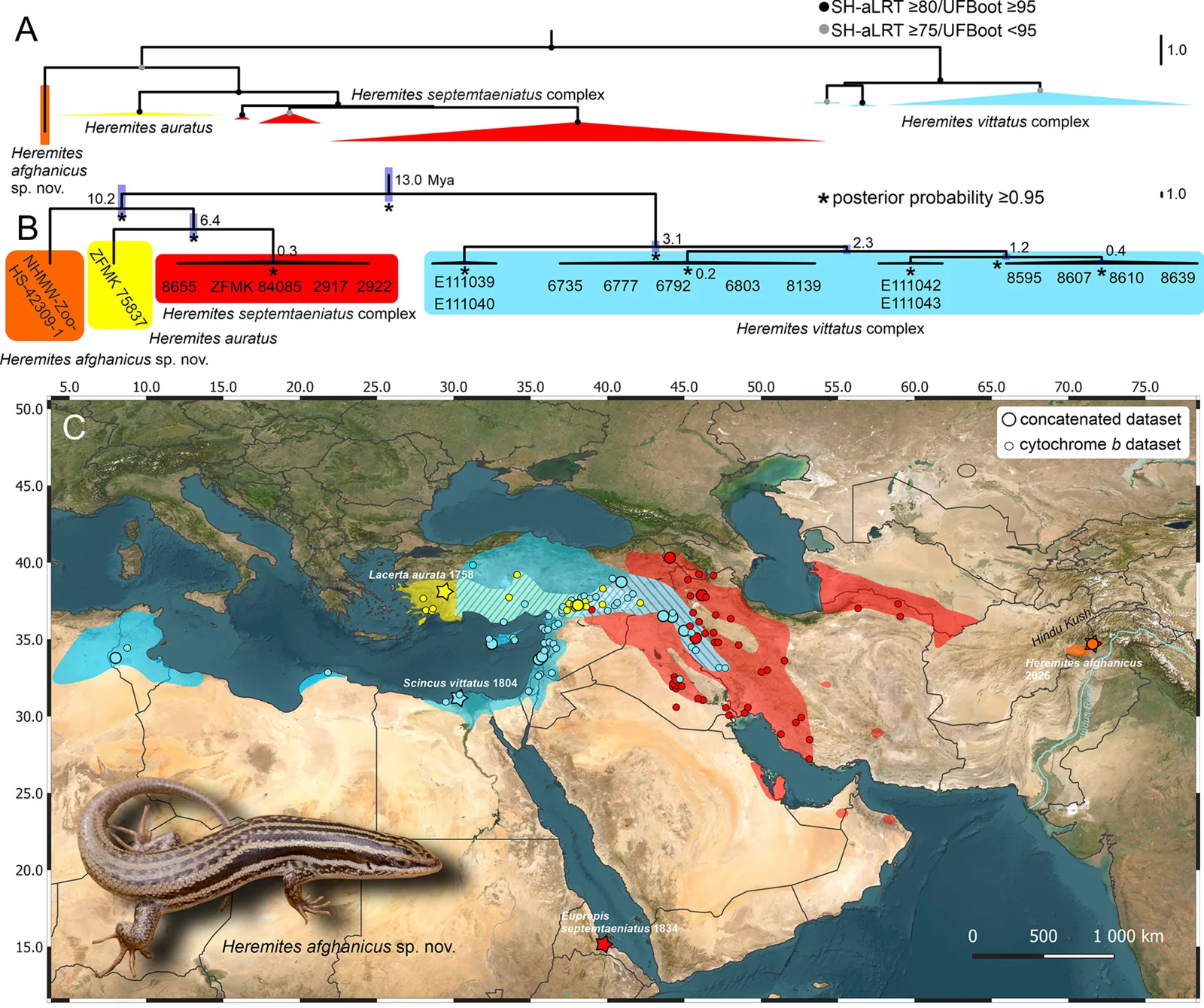
Phylogenetic relationships and geographic distribution of the genus *Heremites*. **A**. Maximum-likelihood phylogeny of the genus based on cytochrome *b*. Black and grey circles indicate SH-aLRT/UFBoot support categories as shown in the panel legend; **B**. Time-calibrated, concatenated (12S, 16S, cyt*b*, ND2, MC1R, RAG1) Bayesian phylogeny of the genus *Heremites* with terminal labels corresponding to sequence or museum voucher IDs. Coloured blocks indicate the four recovered clades: *H. afghanicus* sp. nov. (orange), *H. auratus* (yellow), *H. septemtaeniatus* complex (red), and *H. vittatus* complex (blue). Asterisks on the tree (**B**) denote nodes with posterior probability ≥ 0.95; blue bars indicate 95% highest posterior density (HPD) intervals for the estimated node ages; numbers at selected nodes indicate mean divergence times (Mya); **C**. Approximate distribution of *Heremites* across the eastern Mediterranean, the Middle East, and adjacent regions based on a combination of distribution and genetic data. Coloured polygons show the approximate distributions of the respective lineages in (**A, B**); circles mark localities of genotyped samples included in the phylogeny, and stars indicate type localities. Hatched areas represent inferred zones of overlap/contact between *H. auratus* and the *H. vittatus* complex. Inset shows a live *H. afghanicus* sp. nov. (photograph: Daniel Jablonski).

Maximum-likelihood phylogenetic analyses based on the cyt*b* dataset recovered clear differentiation among the major lineages of *Heremites* and the analysed population from Pakistan. The resulting topology (Fig. 1A) resolved four main lineages corresponding to the deeply divergent Hindu Kush lineage, *H. auratus* (SH-aLRT/UFBoot = 94.0/99), the *H. septemtaeniatus* complex (85.4/96; with additional sublineage structure), and the *H. vittatus* complex (96.6/99), the latter also exhibiting pronounced internal diversity. Collectively, these lineages are distributed, albeit discontinuously, from North Africa to the foothills of the Hindu Kush, and three of them show partial sympatry in southern Turkey (Fig. 1C; Suppl. material 1: fig. S1).

The dated, concatenated Bayesian phylogeny, although based on limited available sampling and sequence data, has a topology similar to that of the cyt*b* tree, recovering the Hindu Kush lineage as a deeply divergent and well-supported clade (posterior probability ≥ 0.95; Fig. 1). Within the genus *Heremites*, the *H. vittatus* complex formed a strongly supported clade that diverged from the clade comprising *H. auratus*, the *H. septemtaeniatus* complex and the new species at ca 13.0 Mya (PP ≥ 0.95). *Heremites auratus* (ZFMK 75837; S Turkey) was recovered as sister to the *H. septemtaeniatus* complex (PP ≥ 0.95) (Armenia, W Iran, E Iraq) with an estimated divergence of ca 6.4 Mya. The lineage leading to *H. afghanicus* sp. nov. diverged from the common ancestor of *H. auratus* and the *H. septemtaeniatus* complex at an estimated 10.2 Mya (Fig. 1B, Suppl. material 1: fig. S2).

Within available concatenated sequences of the *H. septemtaeniatus* complex, sampled terminals showed shallow internal differentiation, with a recent differentiation dated to ca 0.3 Mya (PP ≥ 0.95). In contrast, the *H. vittatus* complex exhibited pronounced internal structure. The earliest split within this complex separated the lineage represented by E111039–E111040 (Tunisia) from the remaining samples at ca 3.1 Mya (PP ≥ 0.95) (the Levant, E Turkey, N Iraq). Subsequent diversification involved several supported nodes, including splits dated to ca 2.3 Mya and 1.2 Mya (PP ≥ 0.95), followed by very shallow divergences among terminal subclades (down to ca 0.4–0.2 Mya; Fig. 1B, Suppl. material 1: fig. S2).

**Figure 2.**
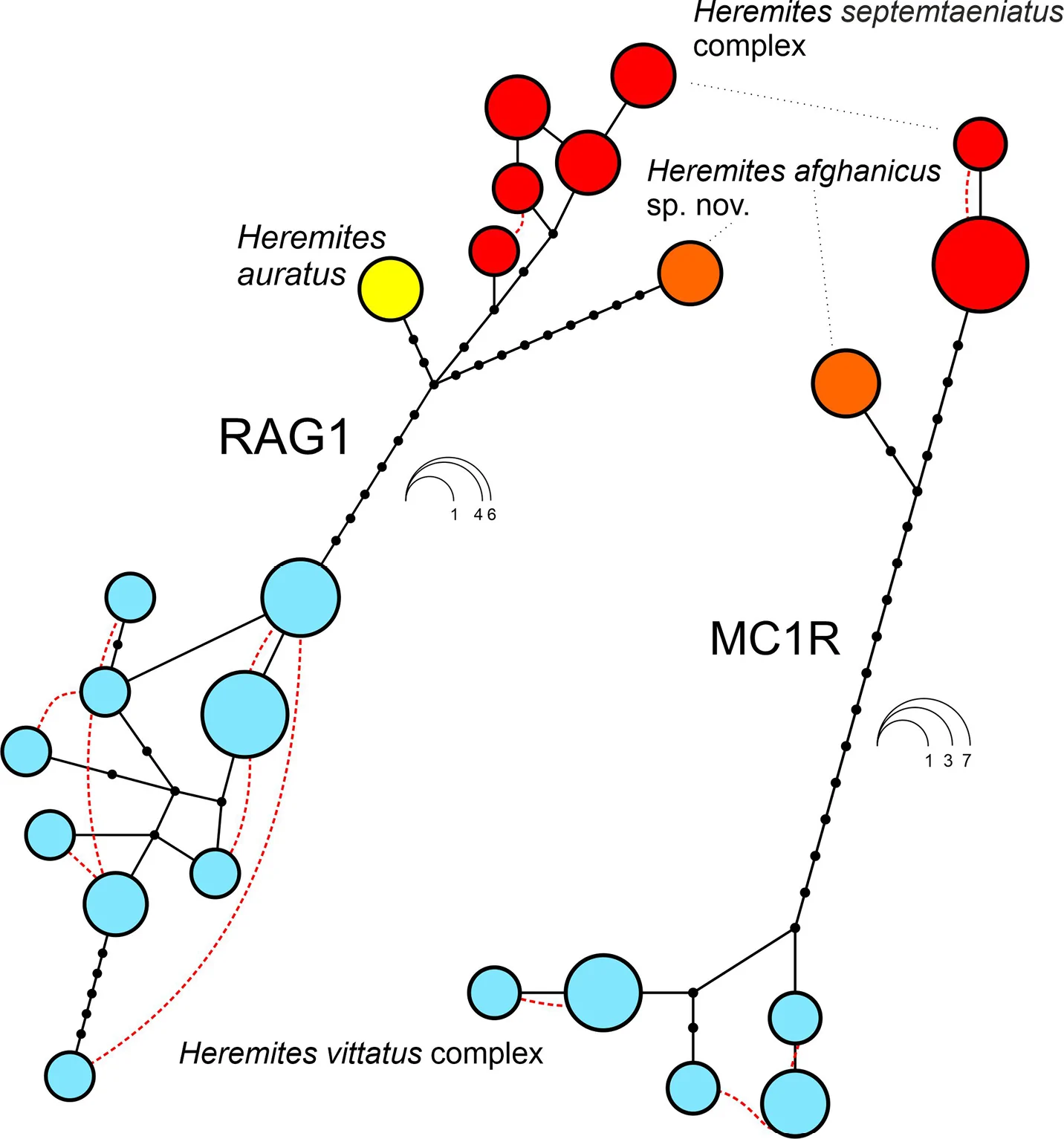
Ninety-five percent statistical parsimony allele networks for the nuclear loci RAG1 and MC1R showing relationships among phased alleles of *Heremites*. Each circle represents a unique allele, and circle size is proportional to allele frequency. Colours indicate lineage/species assignment according to Fig. 1: yellow, *H. auratus*; red, *H. septemtaeniatus* complex; orange, *H. afghanicus* sp. nov.; cyan, *H. vittatus* complex. Solid black lines represent mutational connections inferred by the TCS algorithm, and small black circles represent inferred unsampled alleles. Red dashed lines connect the two phased alleles recovered from the same heterozygous individual.

Allele networks inferred for the nuclear markers RAG1 and MC1R revealed complete allelic differentiation among the sampled major lineages/species of *Heremites* (Fig. 2). In the RAG1 network, the single analysed individual of *H. afghanicus* sp. nov. was represented by a private allele separated by several mutational steps from the alleles of *H. auratus*, the *H. septemtaeniatus* complex, and the *H. vittatus* complex. In the MC1R network, *H. afghanicus* sp. nov. was again represented by a unique allele. No allele sharing was detected among the sampled clades. The single analysed individual of *H. afghanicus* sp. nov. carried private alleles at both nuclear loci. Although the limited sampling precludes assessment of allele frequencies or fixation, these data are consistent with the mitochondrial differentiation of the lineage.

Uncorrected pairwise p-distances indicate high mitochondrial differentiation of the Hindu Kush lineage from western congeners. At cyt*b* (505 bp), the Hindu Kush lineage differed by 15.8% from the *H. septemtaeniatus* complex and by 15.2% from the *H. vittatus* complex, while the distance between the *H. septemtaeniatus* and *H. vittatus* complexes was 14.9%. At ND2 (943 bp), divergence of the Hindu Kush lineage was 17.0% from the *H. septemtaeniatus* complex (GU931546 = ZFMK 84085; W Iran) and 18.6% from *H. auratus* (GU931545 = ZFMK 75837; S Turkey), and distances to the *H. vittatus* complex averaged 20.5% (range 20.2–21.0%); for comparison, ND2 divergence between *H. auratus* and the *H. septemtaeniatus* complex was 11.6%, and the maximum ND2 divergence observed within the *H. vittatus* complex was 6.8%. In contrast, at MC1R (599 bp) the Hindu Kush lineage differed by 1.17% from the *H. septemtaeniatus* complex (KX365022 = ZFMK 84085) and by 2.59% from the *H. vittatus* complex; at RAG1 (523 bp), the new species differed by 3.0% from the *H. septemtaeniatus* complex, 2.5% from *H. auratus* (KX365048 = ZFMK 75837) and 3.3% from the *H. vittatus* complex.

**Figure 3.**
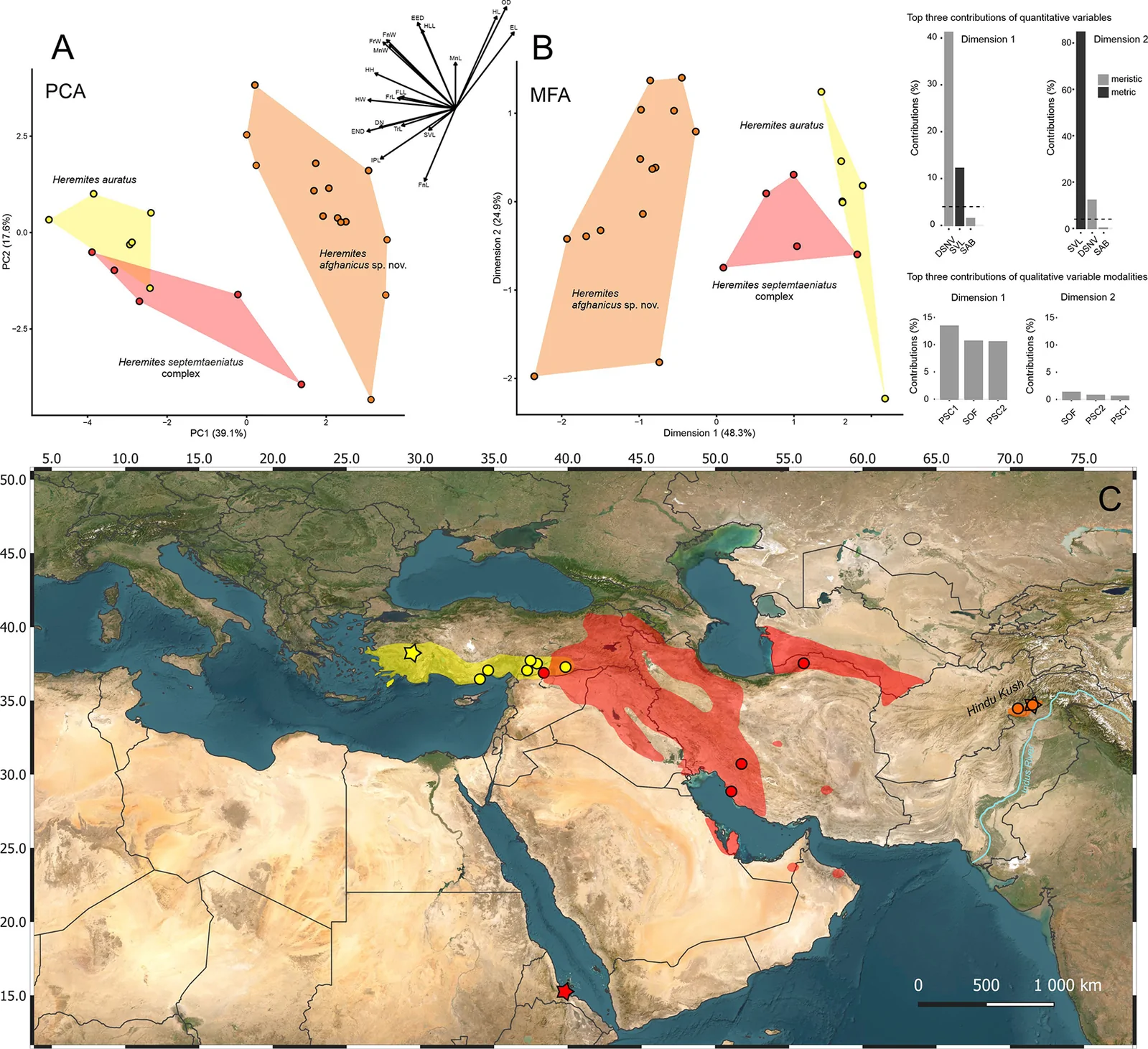
Morphological differentiation and distributional context of *Heremites afghanicus* sp. nov. **A**. Principal component analysis (PCA) based on size-normalised morphometric characters; arrows indicate variable loadings and coloured polygons represent group convex hulls; **B**. Multiple factor analysis (MFA) integrating morphometric, meristic, and categorical characters; coloured polygons represent group convex hulls. Bar plots show the three strongest contributions of quantitative variables and qualitative variable modalities to Dimensions 1 and 2; **C**. Geographic distribution of the compared *Heremites* lineages; circles indicate sampled localities, coloured polygons show approximate distributions, and stars indicate type localities. Colours are consistent across panels: orange, *H. afghanicus* sp. nov.; yellow, *H. auratus*; red, *H. septemtaeniatus* complex.

Multivariate analyses of morphology recovered consistent differentiation of the Hindu Kush lineage from western *Heremites* (Fig. 3). In the PCA, specimens of the Hindu Kush lineage formed a distinct cluster relative to *H. auratus* and the *H. septemtaeniatus* complex, with PC1 and PC2 explaining 39.1% and 17.6% of the total variance, respectively. This pattern was reinforced by the MFA integrating morphometric, meristic, and categorical characters, where Dim1 (48.3%) and Dim2 (24.9%) further separated the Hindu Kush lineage from the compared western lineages. Among quantitative variables, Dim1 was driven predominantly by DSNV, with SVL providing the second-largest contribution and SAB contributing to a lesser extent, whereas Dim2 was dominated by size-related morphometric variation, particularly SVL (Fig. 3B). Notably, one individual of the Hindu Kush lineage and one of *H. auratus* had considerably smaller SVL than the remaining specimens, which may have contributed disproportionately to variation along Dim2.

Thus, based on congruent evidence from our multilocus datasets, morphology, and distribution, we demonstrate that the northwestern Pakistani population represents a distinct evolutionary lineage and we describe it herein as a new species. We further document its known distribution, habitat associations at the Palearctic–Oriental margin and discuss the implications of its discovery for understanding biogeographic structure and hidden diversity in the Hindu Kush region.

### Taxonomy

#### Family Scincidae Oppel, 1811

**Genus *Heremites* Gray, 1845**

##### Heremites afghanicus sp. nov.

Taxon classificationAnimaliaSquamataScincidae

564C4F51-325F-59E3-9647-519C678B3F70

https://zoobank.org/92218DE1-9E3B-43AE-B630-06223800991A

Figs 4, 5

###### Suggested vernacular names.

English name: Afghan grass skink or Dir grass skink.

Pashto name: د افغانستان واښه څرمښکۍ یا دیر واښه څرمښکۍ (Da Afghanistan Wakha Sarmakhkay, Dir Wakha Sarmakhkay).

Urdu name: افغانستان گھاس والی چھپکلی یا دیر گھاس والی چھپکلی (Afghanistan Ghas wali Chipkali, Dir Ghas wali Chipkali).

###### Type material.

**Figure 4.**
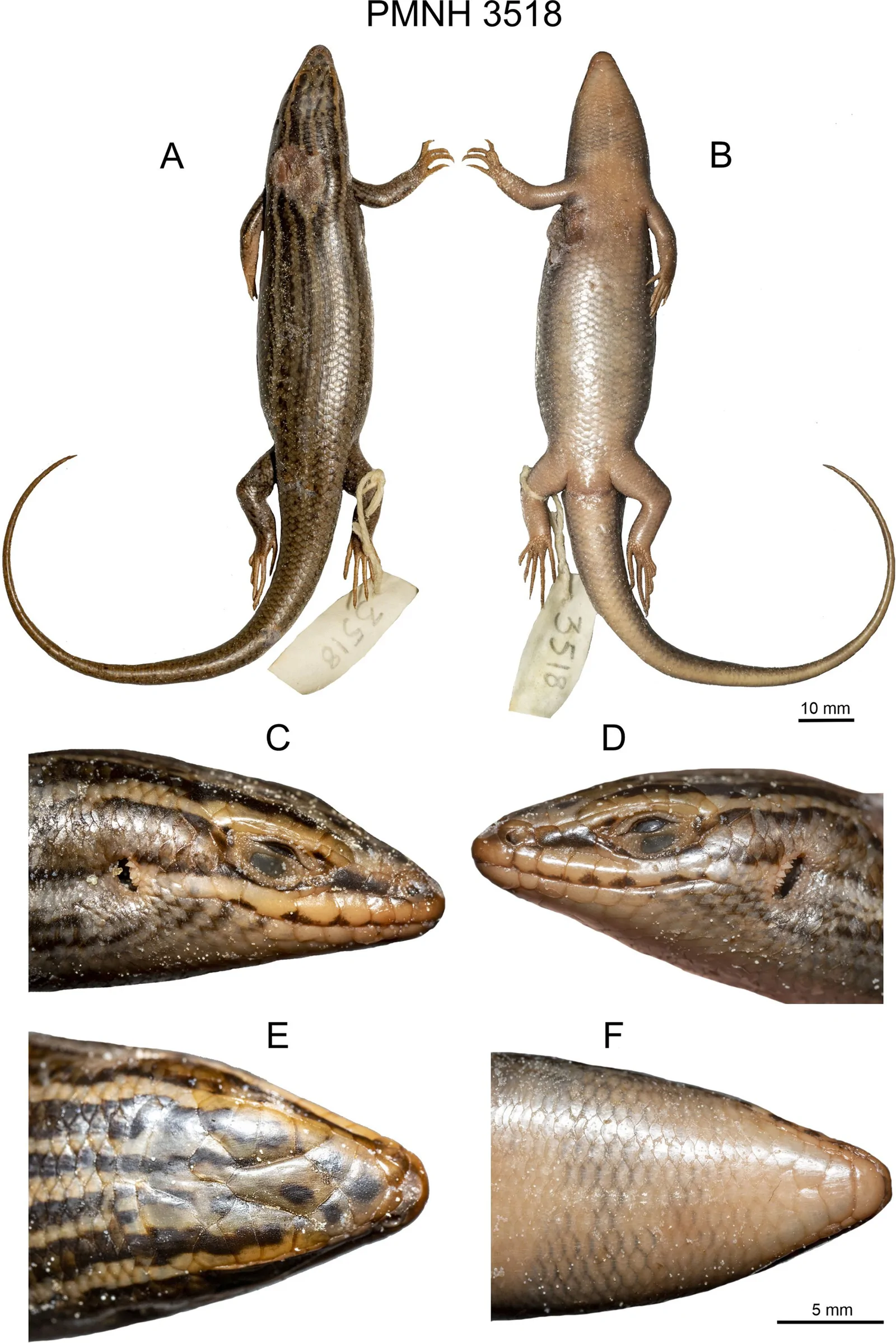
The holotype (adult female) of *Heremites afghanicus* sp. nov. from Shah Alam Baba, Tehsil Adinzai, Lower Dir District, Khyber Pakhtunkhwa Province, Pakistan. **A**. Dorsal view of the body; **B**. Ventral view of the body; **C, D**. Lateral views of the head from both sides; **E**. Dorsal view of the head; **F**. Ventral view of the head. Scale bars as indicated.

***Holotype***. • PMNH 3518 (adult female), Shah Alam Baba, Tehsil Adinzai, Lower Dir District, Khyber Pakhtunkhwa Province, Pakistan (34.7367°N, 72.1021°E; 1,110 m a.s.l.; WGS 84), collected 22 August 2014 by Muhammad Idrees (Fig. 4A–F). ***Paratypes***. • PMNH 3475 (adult male), Shah Alam Baba, Tehsil Adinzai, Lower Dir District, Khyber Pakhtunkhwa Province, Pakistan (34.7367°N, 72.1021°E; 1,110 m a.s.l.; WGS 84), collected 14 June 2013 by Muhammad Idrees; • PMNH 3519 (adult male), PMNH 3522 (adult male), and PMNH 3523 (adult male), same locality, collected 22 August 2014 by Muhammad Idrees; • NHMW-Zoo-HS-42309-1 (adult male), molecularly sampled and sequenced (genes and GenBank accession numbers – 12S: PZ348441; 16S: PZ348454; cyt*b*: PZ334424; ND2: PZ338262; MC1R: PZ661696; RAG1: PZ661709), same locality, collected 18 September 2019 by Muhammad Idrees, R. Masroor, and D. Jablonski. • ZFMK 9064 (adult male; erroneously listed as female in the original catalogue of ZFMK), Dar-e-Nur, vic. Shewa, Nangarhar Province, Afghanistan (34.5558°N, 70.6073°E; 1,200 m a.s.l.), collected on 7 April 1972 by Clas M. Naumann.

###### Additional material.

• PMNH 3474 (adult female), PMNH 3476 (adult male), PMNH 3477 (adult female), PMNH 3478 (adult female), PMNH 3520 (adult female), PMNH 3521 (adult male), PMNH 3524 (subadult female). All from Shah Alam Baba, Tehsil Adinzai, Lower Dir District, Khyber Pakhtunkhwa Province, Pakistan.

###### Diagnosis.

*Heremites afghanicus* sp. nov. (southern foothills of the Hindu Kush; currently known from NW Pakistan and E Afghanistan) can be distinguished from the western Palearctic congeners compared here (*H. auratus*, *H. septemtaeniatus* complex, *H. vittatus* complex) by a unique combination of molecular divergence and external morphology. It represents a deeply divergent lineage in mitochondrial and nuclear DNA, differing from the compared western congeners by uncorrected p-distances of 15.2–15.8% in cyt*b* (505 bp) and 17.0–21.0% in ND2 (943 bp), while showing lower but still remarkable and consistent divergence in the nuclear markers (MC1R = 1.17–2.59%; RAG1 = 2.5–3.3%). Morphologically, it is diagnosable by the following combination of characters (type series and additional material, *n* = 14, Table 1; unless stated otherwise): (1) parietal shields in contact (PSC present in 14/14); (2) contact between the third supraocular and the frontal (SOF present in 14/14); (3) prefrontals usually in contact (PFC present in 10/14); (4) supralabials six or seven (mode 7), infralabials 6–8 (mode 7); (5) 34–38 scales around midbody (SAB), 52–60 dorsal scales from the first nuchal to the level above the vent (DSNV), and 60–69 ventral scales in a longitudinal series from chin shields to cloaca (VT); (6) subcaudals 78–98 (SC); (7) ear opening with 3–5 projecting scales (ear pectination, EP; mode 4); (8) dorsal scales consistently with three keels (KDS = 3); (9) comparatively short eye–nostril distance (END 2.7–5.1 mm), especially relative to *H. auratus* and the *H. septemtaeniatus* complex. Furthermore, *H. afghanicus* sp. nov. differs from the specimens assigned to the *H. septemtaeniatus* complex in exhibiting higher HL/HW ratios. In multivariate space based on combined metric, meristic, and categorical characters, individuals of *H. afghanicus* sp. nov. form a discrete cluster relative to *H. auratus* and the *H. septemtaeniatus* complex (MFA: Dim1 = 48.3%, Dim2 = 24.9% of total variance; PCA on metric data: PC1 = 39.1%, PC2 = 17.6%).

**Table 1.** Descriptive statistics for morphometric, meristic, and qualitative characters in the genus *Heremites*. For each taxonomic group, values are given as mean ± SD (minimum–maximum) followed by sample size (*n*), except HL/HW, which is given as minimum–maximum. Morphometric measurements (in mm) were taken following the definitions provided in the Materials and methods; scalation and other meristic characters are expressed as counts. Statistics were calculated from all available specimens per character; therefore, n may differ among characters due to missing data. Qualitative characters (SOF, PFC, PSC, white spots) are summarised separately as state frequencies.

**Character**	***Heremites afghanicus* sp. nov**.	***Heremites auratus***	***Heremites septemtaeniatus* complex**	***Heremites vittatus* complex**
**Morphometric data (mm)**				
SVL	73.8 ± 16.2 (38.8–92.3), *n* = 14	84.6 ± 13.6 (59.1–99.6), *n* = 6	76.4 ± 8.2 (65.5–88.6), *n* = 6	79.7 ± 2.1 (78.3–81.2), *n* = 2
TL	95.3 ± 20.1 (66.4–111.7), n = 7	110.2 ± 14.1 (89.4–121.0), *n* = 4	85.2 ± 22.3 (63.7–114.5), *n* = 4	–
HL	15.9 ± 2.8 (8.5–19.0), *n* = 14	15.1 ± 2.0 (11.2–16.8), *n* = 6	13.4 ± 2.5 (8.8–16.4), *n* = 7	14.6 ± 0.5 (14.3–15.0), *n* = 2
HW	9.5 ± 1.6 (5.1–11.0), *n* = 14	12.6 ± 2.0 (8.9–14.7), *n* = 6	10.8 ± 1.6 (9.2–13.7), *n* = 7	8.9 ± 0.2 (8.7–9.1), *n* = 2
HL/HW	1.45–1.84	1.03–1.29	0.89–1.50	1.64–1.65
HH	7.3 ± 1.5 (3.6–8.7), *n* = 14	8.6 ± 1.0 (6.8–9.6), *n* = 6	7.7 ± 1.1 (6.7–9.4), *n* = 7	6.9 ± 0.1 (6.8–6.9), *n* = 2
TrL	36.4 ± 9.7 (17.4–49.1), *n* = 14	39.9 ± 7.3 (27.3–50.2), *n* = 6	35.7 ± 3.4 (32.7–41.4), *n* = 6	–
OD	2.6 ± 0.5 (1.1–3.0), *n* = 14	2.1 ± 0.3 (1.6–2.4), *n* = 6	1.9 ± 0.4 (1.5–2.6), *n* = 7	–
EL	1.8 ± 0.5 (0.7–2.4), *n* = 14	1.4 ± 0.2 (1.2–1.8), *n* = 6	1.2 ± 0.1 (1.0–1.4), *n* = 7	1.5 ± 0.0 (1.4–1.5), *n* = 2
DN	2.2 ± 0.5 (1.3–3.0), *n* = 14	2.8 ± 0.6 (1.9–3.5), *n* = 6	2.5 ± 0.5 (1.9–3.4), *n* = 7	–
END	4.2 ± 0.6 (2.7–5.1), *n* = 14	6.4 ± 1.1 (4.4–7.6), *n* = 6	6.2 ± 0.8 (5.6–7.8), *n* = 7	3.6 ± 0.3 (3.3–3.8), *n* = 2
EED	4.8 ± 0.8 (3.0–5.6), *n* = 14	5.0 ± 0.7 (3.9–5.8), *n* = 6	4.6 ± 0.7 (4.0–5.9), *n* = 7	4.8 ± 0.3 (4.6–5.1), *n* = 2
FrW	2.6 ± 0.5 (1.3–3.1), *n* = 14	2.8 ± 0.3 (2.3–3.1), *n* = 6	2.6 ± 0.2 (2.3–3.0), *n* = 7	2.6 ± 0.0 (2.6–2.7), *n* = 2
FrL	4.6 ± 0.7 (3.1–5.5), *n* = 14	4.9 ± 0.8 (3.3–5.5), *n* = 6	4.9 ± 0.7 (4.2–6.4), *n* = 7	–
FnW	3.4 ± 0.6 (2.0–4.0), *n* = 14	3.8 ± 0.5 (2.9–4.4), *n* = 6	3.5 ± 0.5 (2.9–4.5), *n* = 7	–
FnL	2.2 ± 0.9 (1.1–5.0), *n* = 14	2.5 ± 0.3 (2.0–2.9), *n* = 6	2.4 ± 0.3 (2.0–2.8), *n* = 7	–
LorWa	1.1 ± 0.6 (0.6–3.0), *n* = 13	0.9 ± 0.0 (0.8–0.9), *n* = 2	1.0 ± NA (1.0–1.0), *n* = 1	–
LorWp	1.6 ± 0.4 (0.9–2.1), *n* = 12	1.1 ± 0.0 (1.1–1.1), *n* = 2	0.8 ± NA (0.8–0.8), *n* = 1	–
LL	1.1 ± NA (1.1–1.1), *n* = 1	1.3 ± 0.1 (1.2–1.4), *n* = 4	1.5 ± 0.3 (1.2–1.8), *n* = 3	–
IPL	2.6 ± 0.5 (1.7–3.6), *n* = 14	3.6 ± 0.6 (3.0–4.7), *n* = 6	3.2 ± 0.5 (2.6–4.1), *n* = 6	–
MnW	3.2 ± 0.7 (1.9–4.0), *n* = 14	3.6 ± 0.5 (2.7–4.2), *n* = 6	3.3 ± 0.5 (2.7–4.2), *n* = 7	–
MnL	1.7 ± 0.3 (0.9–2.0), *n* = 14	1.6 ± 0.2 (1.4–2.0), *n* = 6	1.6 ± 0.2 (1.3–1.9), *n* = 7	–
HLL	30.8 ± 5.1 (18.6–36.1), *n* = 14	31.2 ± 4.7 (22.0–35.3), *n* = 6	29.5 ± 3.4 (23.6–33.8), *n* = 7	25.1 ± 0.2 (25.0–25.2), *n* = 2
FLL	21.8 ± 3.9 (12.4–26.3), *n* = 14	22.8 ± 3.7 (16.1–26.7), *n* = 6	22.9 ± 3.1 (17.7–26.3), *n* = 7	17.8 ± 0.5 (17.4–18.2), *n* = 2
**meristic data**				
SL	6.9 ± 0.4 (6–7), *n* = 14	7.3 ± 0.3 (7–8), *n* = 6	6.9 ± 0.2 (6–7), *n* = 7	7.0 ± 0.0 (7–7), *n* = 2
IL	7.1 ± 0.3 (6–8), *n* = 14	7.4 ± 0.4 (7–8), *n* = 6	7.0 ± 0.0 (7–7), *n* = 7	7.0 ± 0.0 (7–7), *n* = 2
SSLE	3.8 ± 0.4 (3–4), *n* = 14	3.5 ± 0.6 (3–5), *n* = 6	3.3 ± 0.7 (3–5), *n* = 5	
PN	1.1 ± 0.3 (1–2), *n* = 14	1.0 ± 0.0 (1–1), *n* = 6	1.2 ± 0.4 (1–2), *n* = 6	–
SAB	35.5 ± 1.0 (34–38), *n* = 14	37.0 ± 0.0 (37–37), *n* = 6	35.0 ± 1.8 (32–37), *n* = 6	–
DSNV	54.6 ± 2.3 (52–60), *n* = 14	60.5 ± 2.9 (58–66), *n* = 6	60.4 ± 3.1 (57–65), *n* = 5	–
VT	64.6 ± 2.5 (60–69), *n* = 14	64.5 ± 3.8 (57–67), *n* = 6	63.3 ± 2.4 (60–65), *n* = 4	–
SC	88.3 ± 7.3 (78–98), *n* = 7	80.8 ± 11.3 (70–96), n = 4	70.8 ± 9.7 (63–83), *n* = 4	–
EP	4.1 ± 0.7 (3–5), *n* = 14	3.5 ± 1.1 (2–5), *n* = 5	2.4 ± 0.8 (1–3), *n* = 7	–
KDS	3.0 ± 0.0 (3–3), *n* = 14	3.0 ± 0.0 (3–3), *n* = 6	3.0 ± 0.0 (3–3), *n* = 7	–
**qualitative (counts/available)**				
SOF	yes 14/14	no 5/6; yes 1/6	yes 7/7	–
PFC	yes 10/14; no 4/14	no 5/6; yes 1/6	no 6/7; yes 1/7	–
PSC	yes 14/14	no 6/6	no 6/7; not available 1/7	–
WS	yes 1/1	yes 6/6	not visible 3/7; yes 2/7; no 2/7	–

###### Description of holotype.

Specimen preserved in formaldehyde and stored in ethanol. Body elongated and moderately robust, with well-developed pentadactyl limbs and a distinct but weakly constricted neck (Fig. 4A, B). Snout–vent length 80.0 mm; tail complete, long (TL 110 mm) and evenly tapering; trunk length 43.4 mm. Head slightly longer than wide (HL 18.0 mm, HW 10.7 mm) and moderately high (HH 8.4 mm); orbit relatively large (OD 2.9 mm); ear opening distinct (EL 2.1 mm) with moderate ear pectination (EP = 4; Fig. 4C, D). Distances across the head: DN 2.0 mm, END 4.4 mm, EED 5.2 mm. Dorsal head shields well developed; frontal longer than wide (FrL 5.0 mm, FrW 2.8 mm) and frontonasal wider than long (FnW 3.7 mm, FnL 2.3 mm). Loreal region with anterior (LorWa 1.2 mm) and posterior loreals (LorWp 1.5 mm) present. Interparietal length 2.9 mm; mental width 3.2 mm and length 1.6 mm. Forelimb length 23.4 mm; hindlimb length 33.5 mm. Scalation of the head and trunk typical for the genus: supralabials 7; infralabials 7; four scales between the last supralabial and the ear opening (SSLE = 4); one pair of nuchals (PN = 1). Contact between the third supraocular and frontal present (SOF: yes); prefrontals in contact (PFC: yes); parietal shields in contact (PSC: yes). Midbody scale rows (SAB) 36; 56 dorsal scales from the first nuchal to the level above the vent (DSNV = 56); 69 ventral scales in a longitudinal series from chin shields to cloaca (VT = 69). Subcaudals 87 (SC = 87). Dorsal scales with three keels (KDS = 3).

Dorsal part dark brown to olive-brown with a well-defined longitudinal pattern, consisting of darker stripes and paler interspaces that produce a distinctly striped appearance (Fig. 4A). Head dorsum mottled brown with paler longitudinal elements; a pale stripe extends from the snout through the orbital region towards the temporal area, contrasting with darker pigmentation on the upper temporal region (Fig. 4C–E). Labial scales pale beige to light brown. Flanks darker than venter, with subtle speckling. Ventral surfaces (throat, belly, underside of tail) uniform pale greyish beige with minimal patterning (Fig. 4B, F). Presence of white spots was not recorded for this specimen.

###### Description of paratypes.

**Figure 5.**
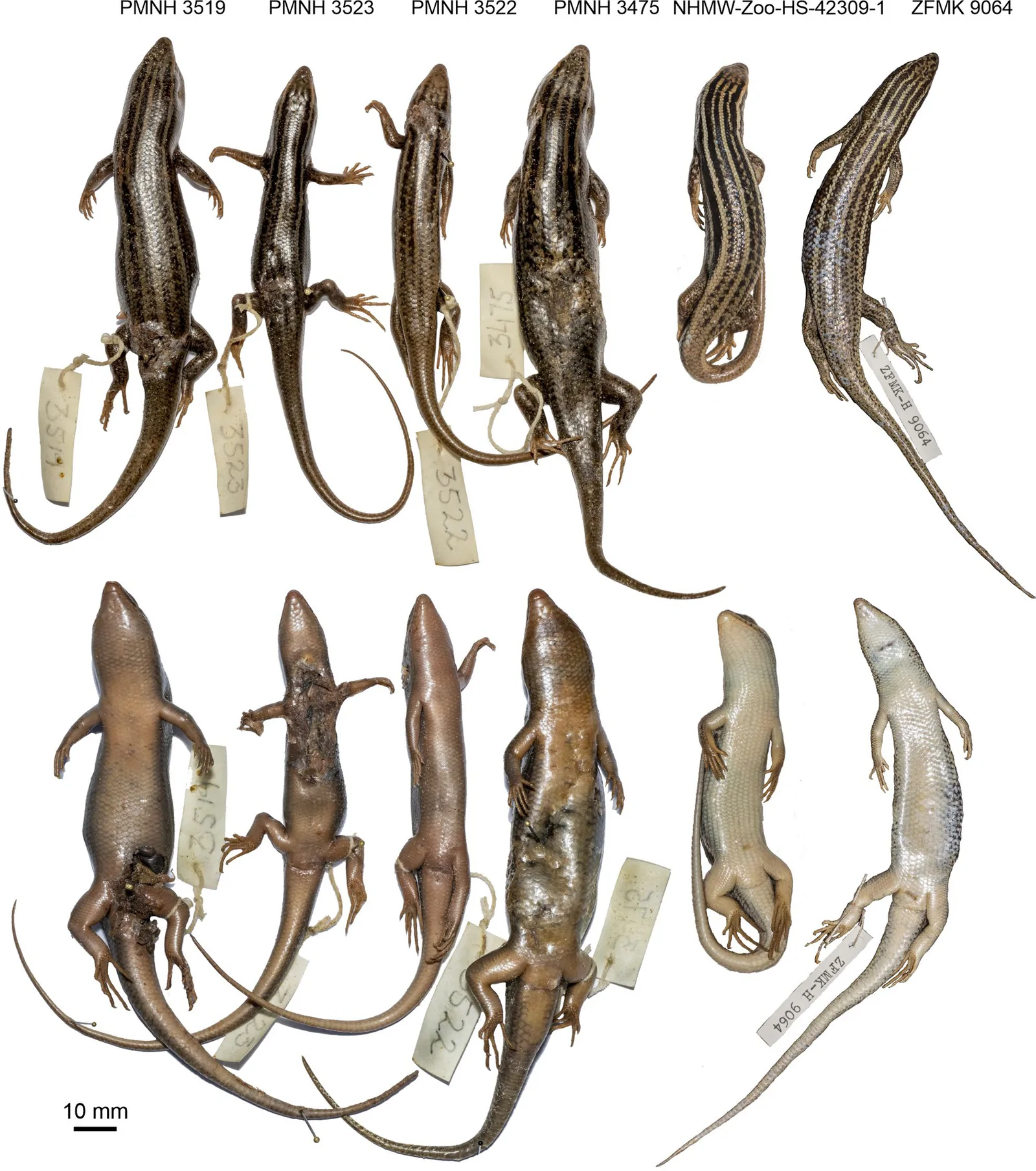
Paratypes of *Heremites afghanicus* sp. nov. from Shah Alam Baba, Tehsil Adinzai, Lower Dir District, Khyber Pakhtunkhwa Province, Pakistan (PMNH 3475, 3519, 3522, 3523, NHMW-Zoo-HS-42309-1) and Dar-e-Nur, vic. Shewa, Nangarhar Province, Afghanistan (ZFMK 9064).

The paratypes of *Heremites afghanicus* sp. nov. correspond to the holotype (Fig. 4) in overall morphology, body proportions, and colour pattern (Fig. 5), with the differences summarised in Table 2. All paratypes are adult males. Snout–vent length (SVL) ranges from 51.7 to 92.3 mm. Tail length (TL) ranges from 68.0 to 111.7 mm; tail regeneration is recorded in PMNH 3519 and PMNH 3475. Meristic variation among paratypes is limited. Supralabials vary between 6 and 7 (SL = 6 in ZFMK 9064 and PMNH 3475; SL = 7 in the remaining paratypes). Infralabials range from 6 to 8 (typically 7; IL = 8 in ZFMK 9064; IL = 6 in NHMW-Zoo-HS-42309-1). The number of scales between the last supralabial and the ear opening ranges from 3 to 4 (SSLE = 3 in NHMW-Zoo-HS-42309-1 and PMNH 3475; SSLE = 4 in the remaining paratypes). One pair of nuchals is constant (PN = 1). Scales around midbody range from 35 to 38 (SAB = 38 in NHMW-Zoo-HS-42309-1; 35–36 in the remaining paratypes). Dorsal scale counts from the first nuchal to the level above the vent range from 52 to 60 (DSNV = 52–60), and ventral scales range from 62 to 68 (VT = 62–68). Subcaudals range from 78 to 98 (SC = 78–98). Ear pectination varies between four and five (EP = 4–5). The contact between the third supraocular and the frontal is present in all paratypes (SOF present), and parietal shields are consistently in contact (PSC present). In contrast, prefrontals vary, being in contact in most paratypes but separated in ZFMK 9064 and PMNH 3522. All paratypes have dorsal scales with three keels (KDS = 3). White spots were recorded in the paratype NHMW-Zoo-HS-42309-1 but were not observed or not recorded in the remaining paratypes.

**Table 2.** Morphometric, meristic, and qualitative data of the type series of *Heremites afghanicus* sp. nov. For abbreviations of characters, see Materials and methods (rg = regenerated, NA = not available). All morphometric measurements are in mm.

**Voucher number**	***PMNH 3518 holotype***	***NHMW-Zoo-HS-42309-1 paratype***	***PMNH 3475 paratype***	***PMNH 3519 paratype***	***PMNH 3522 paratype***	***PMNH 3523 paratype***	***ZFMK 9064 paratype***
Country	Pakistan	Pakistan	Pakistan	Pakistan	Pakistan	Pakistan	Afghanistan
age	adult	adult	adult	adult	adult	adult	adult
sex	F	M	M	M	M	M	M
SVL	80.0	51.7	92.3	79.6	62.5	63.7	92.0
TL	110	68.0	91.5 (rg)	93.1 (rg)	93.9	111.7	105.8
HL	18.0	11.58	19.0	17.3	15.3	15.3	16.5
HW	10.7	7.94	10.3	10.0	9.0	8.7	11.0
HH	8.4	6.1	8.5	8.0	6.3	6.2	8.1
TrL	43.4	22.8	45.2	37.1	30.1	29.2	49.1
OD	2.9	1.9	3.0	2.7	2.5	2.5	2.8
EL	2.1	1.4	2.4	1.8	1.4	1.5	2.2
DN	2.0	2.1	3.0	2.0	2.0	2.0	2.5
END	4.4	5.1	5.0	4.2	3.8	3.8	4.1
EED	5.2	3.6	5.6	5.1	4.3	4.4	5.0
FrW	2.8	2.0	3.1	2.7	2.3	2.2	2.9
FrL	5.0	3.6	5.2	5.1	4.4	4.0	5.5
FnW	3.7	3.0	4.0	3.5	3.0	3.0	3.1
FnL	2.3	1.5	2.3	2.4	2.2	1.7	5.0
LorWa	1.2	NA	1.1	1.0	0.8	0.9	1.1
LorWp	1.5	NA	2.0	1.4	1.5	1.3	2.1
LL	NA	1.1	NA	NA	NA	NA	NA
IPL	2.9	2.4	3.0	2.7	2.1	2.2	3.6
MnW	3.2	2.6	4.0	3.3	2.6	2.7	3.0
MnL	1.6	1.4	2.0	1.7	1.6	1.6	2.0
HLL	33.5	24.3	36.0	32.1	29.3	28.2	35.0
FLL	23.4	16.0	25.8	23.8	20.3	20.5	25.1
SL	7	7	6	7	7	7	6
IL	7	6	7	7	7	7	8
SSLE	4	3	3	4	4	4	4
PN	1	1	1	1	1	1	1
SAB	36	38	35	35	35	36	35
DSNV	56	60	54	56	52	53	54
VT	69	65	62	66	65	68	62
SC	87	78	82 (rg)	72 (rg)	86	98	86
EP	4	5	4	4	4	4	5
SOF	yes	yes	yes	yes	yes	yes	yes
PFC	yes	yes	yes	yes	no	yes	no
PSC	yes	yes	yes	yes	yes	yes	yes
KDS	3	3	3	3	3	3	3
WS (white spots)	NA	yes	NA	NA	NA	NA	NA

###### Variation.

Variation in the series of *H. afghanicus* sp. nov. (NHMW-Zoo-HS-42309-1, PMNH 3474-3478, 3518-3524, ZFMK 9064; *n* = 14) is primarily expressed in body and tail size, and several head-scale contacts/counts. Snout–vent length ranges from 38.8 to 92.3 mm (mean 73.8). Complete tail length ranges from 66.4 to 111.7 mm (mean 95.3), and subcaudal counts in specimens with complete tails range from 78 to 98 (mean 88.3). Head dimensions vary as follows: HL 8.5–19.0 mm, HW 5.1–11.0 mm, HH 3.6–8.7 mm. Trunk length ranges from 17.4 to 49.1 mm. Head scalation is relatively stable, with six or seven supralabials (usually 7) and 6–8 infralabials (usually 7), and three or four scales between the last supralabial and the ear opening (mode 4). The number of nuchal pairs varies between one and two. The contact between the third supraocular and frontal is constant (present in all examined specimens). The parietal shields are also consistently in contact (present in all examined specimens) in contrast to the *H. septemtaeniatus* complex (including *H. s. transcaucasicus*) in which they are separated (Anderson 1999). In contrast, prefrontals are variable, being in contact in most individuals (10/14) but separated in some (4/14). Ear pectination varies between three and five projecting scales (mode 4). Trunk scalation shows moderate variation: 34–38 scales around midbody, 52–60 dorsal scales from the first nuchal to the level above the vent, and 60–69 ventral scales from chin shields to cloaca. Dorsal scales consistently bear three keels (no variation observed). Presence of white spots was recorded as present in the single specimen where this character was scored (1/1) and should be re-evaluated across additional material.

###### Comparison.

*Heremites afghanicus* sp. nov. differs from *H. auratus* by the constant presence of parietals in contact; the parietals in *H. auratus* are, however, separated. Contact between the third supraocular and the frontal was present in all examined *H. afghanicus* sp. nov. (14/14), compared with one of six examined *H. auratus*. Prefrontal contact was also more frequent in *H. afghanicus* sp. nov. (10/14) than in the examined *H. auratus* (1/6). *Heremites afghanicus* sp. nov. generally has the lowest number of dorsal scales (52–60) from the first nuchal to the level above the vent (DSNV) vs 58–66 in *H. auratus*. *Heremites afghanicus* sp. nov. has a shorter END (2.7–5.1 mm) vs *H. auratus* (4.4–7.6 mm). The new species differs from the *H. septemtaeniatus* complex by the constant parietal contact (PSC present in 14/14 vs absent in all scored specimens of the *H. septemtaeniatus* complex), higher EP counts (3–5 vs 1–3), shorter END (2.7–5.1 vs 5.6–7.8 mm), and lower DSNV (52–60 vs 57–65). The separation of the phylogenetic group comprising *H. afghanicus* sp. nov. from the *H. vittatus* complex is supported primarily by the deep phylogenetic divergence based on the concatenated mitochondrial and nuclear DNA data, large genetic distances, clearly distinctive colouration and pattern and geographic isolation from other members of the genus.

###### Distribution and ecology.

Currently, *H. afghanicus* sp. nov. is known only from two localities, one in Afghanistan and the second in Pakistan (Moravec et al. 2006; Masroor et al. 2021). The type series of *H. afghanicus* sp. nov. thus originates from two foothill localities on the southern margin of the Hindu Kush. The holotype, together with Pakistani paratypes, was collected in Lower Dir District (Khyber Pakhtunkhwa, northwestern Pakistan), a montane landscape with an elevation from 1,100 to 1,400 m a.s.l., where elevation declines southwards along the Panjkora River and vegetation forms a mosaic of moist temperate forest, subtropical Chir pine, and subtropical broad-leaved forest (see photo of the location in Masroor et al. 2021). The local climate is temperate, with cold winters and warm to hot, humid summers influenced by monsoonal rainfall. At the type locality, individuals were recorded on a rocky, hilly slope with large boulders and outcrops providing basking sites and crevice refugia, surrounded by mixed woody vegetation including *Monotheca buxifolia*, *Eucalyptus camaldulensis*, *Ficus carica*, *Ailanthus altissima*, *Olea ferruginea*, and *Morus* spp., with shrubs dominated by *Dodonaea viscosa*, *Rumex hastatus*, and *Indigofera heterantha*, and a grass-rich ground layer (*Apluda mutica*). The morphologically assigned paratype (ZFMK 9064) originates from Dar-e-Nur, vic. Shewa, Nangarhar Province, eastern Afghanistan, at ca 1,200 m a.s.l. This area represents a rugged valley-and-slope setting in the monsoon-influenced foothills northeast of Jalalabad, characterised by a heterogeneous woodland-to-open habitat mosaic typical of the eastern Afghan forest belt. At this elevation, evergreen oak woodland is expected to be prominent locally, interspersed with rocky slopes, ravines, and patches of human-modified habitat, providing a structurally complex environment with abundant rock cover and suitable microhabitats for basking and shelter. The two localities are ~140 km apart in a straight line. Based on the two currently known occurrence localities, the documented area of occupancy (AOO), calculated using a standard 2 × 2 km grid, is 8 km^2^, corresponding to two occupied grid cells.

###### Etymology.

The specific epithet afghanicus is a Latinised adjective (masculine nominative singular) derived from “Afghan-” with the Latin suffix -*icus* (pertaining to or originating from). The name refers not only to Afghanistan in a strict geopolitical sense, but explicitly to Afghans (Pashtuns), i.e., the Pashto-speaking ethnic group associated with the southern slopes of the Hindu Kush and adjacent regions. In the same sense, the historical toponym **“**Afghania**”** (used in older geographical/historical contexts) underlies several regional designations connected with Afghans/Pashtuns, including the broader area corresponding to today’s Khyber Pakhtunkhwa in Pakistan and adjoining transboundary Hindu Kush foothills. By analogy, *H. afghanicus* sp. nov. highlights the species’ geographic affiliation with the Pashtun-dominated belt along the southern Hindu Kush, reflecting this biogeographic and cultural-historical linkage.

## Discussion

Our study substantially improves our understanding of the long-standing uncertainties in phylogenetic relationships and taxonomy in the genus *Heremites* (Baier et al. 2017; Bahmani et al. 2018) and sheds light on a previously enigmatic and taxonomically unresolved population from the Hindu Kush, collected in 1972 but officially reported 20 years ago from Afghanistan (Moravec et al. 2006) and five years ago from Pakistan (Masroor et al. 2021). Although denser geographic sampling and higher genomic resolution will be necessary to more precisely delimit species and distribution boundaries, quantify range-wide genetic diversity, and reconstruct the evolutionary history of the genus, our data support the newly described species as an old, deeply divergent lineage, distinct genetically, morphologically, and geographically. The time-calibrated concatenated phylogeny presented here suggests a Miocene divergence between the lineage leading to *H. afghanicus* sp. nov. and the ancestor of *H. auratus* and the *H. septemtaeniatus* complex (Fig. 1B), whereas much of the subsequent diversification within the western lineages occurred later within the crown of the genus. This temporal pattern is generally consistent with historical speciation within *Heremites* and spatial isolation of other regional reptiles (e.g. Agarwal et al. 2014, 2022; Bahmani et al. 2018; Ghaedi et al. 2021; Pola et al. 2024; Jablonski et al. 2026) and may also suggest a broader pattern of east–west lineage differentiation coupled with ecological and topographic heterogeneity across the genus’ range, which should be explicitly tested in future studies.

The new species is separated from the nearest known western populations of *Heremites* by more than 700 km (Masroor et al. 2021; Fig. 1C) and currently represents the easternmost known lineage of the genus. Nevertheless, substantial portions of the genus’ distribution remain unsampled (Sindaco and Jeremchenko 2008; Güçlü et al. 2014; Bahmani et al. 2018; Masroor et al. 2021), and further work is thus needed to complete the biogeographic and taxonomic picture. Areas with suitable habitats in central and western Afghanistan should be surveyed to evaluate whether a distribution gap exists between the easternmost Hindu Kush populations and those in central Iran and Turkmenistan. A key remaining question is thus whether *H. afghanicus* sp. nov. is truly isolated (currently known from only two localities; Moravec et al. 2006; Masroor et al. 2021) or represents a more broadly distributed species along the foothills of the Hindu Kush. However, socio-political constraints and harsh environmental conditions across parts of the Hindu Kush in Afghanistan and Pakistan make targeted fieldwork still difficult in the near term (Jablonski et al. 2021). Against this background, our discovery represents a significant step towards understanding the poorly investigated biodiversity of the region between the Indus River and the Hindu Kush mountains.

Masroor et al. (2021) explicitly questioned whether the Hindu Kush population represents a relict population of the *H. septemtaeniatus* complex or an unrecognised lineage within *Heremites*. Our results provide clear evidence that this population constitutes a distinct species (Figs 1, 2, 3), underscoring the need for further genus-wide taxonomic revision based on denser sampling and genomic data. Importantly, the original allocation of the Hindu Kush material to the *H. septemtaeniatus* complex (linked to the name *H. s. transcaucasicus*) was understandable under a morphology-based framework, because several characters commonly emphasised in traditional diagnoses are compatible with that complex, such as the frequent contact between the third supraocular and the frontal (SOF) and the general configuration of head scalation. At the same time, Masroor et al. (2021) highlighted a non-trivial character mosaic that did not match expectations based on western populations, most notably the condition of the parietal shields (PSC) and the overall combination of head-scale contacts and counts, suggesting that the Hindu Kush population may not fit cleanly within any described western taxon. In this context, our multilocus phylogenetic results, together with the multivariate analysis of morphological variation, clarify the placement of this disjunct population and suggest that its mosaic morphology is consistent with long-term isolation rather than with the intraspecific variation documented elsewhere within the *H. septemtaeniatus* complex (Akhmedov and Szczerbak 1987; Faizi and Rastegar-Pouyani 2006; Bahmani et al. 2018).

Because our concatenated dataset includes both mitochondrial and nuclear loci, the placement of *H. afghanicus* sp. nov. is well supported (Fig. 1B). However, comparative nuclear data remain unavailable for many populations across the broad ranges of the *H. septemtaeniatus* and *H. vittatus* complexes (Fig. 1A, C), including several deeply divergent mitochondrial lineages reported from Iran and adjacent regions (Bahmani et al. 2018). As a result, it remains difficult to evaluate the extent to which mitochondrial structure reflects species boundaries versus historical introgression or incomplete lineage sorting across the genus. Expanding nuclear or genomic sampling across these regions will therefore be essential for a robust, range-wide integrative revision and, particularly, to determine the phylogenetic position of populations from Eritrea, including the type locality (Massawa) for *H. septemtaeniatus*.

Nevertheless, the available evidence confirms that deep mitochondrial structuring is present within the *H. septemtaeniatus* complex (Fig. 1A, Suppl. material 1: fig. S1). Bahmani et al. (2018) reported substantial intraspecific diversity in Iranian populations (notably from Khorasan, Isfahan, Khuzestan, and Fars) and designated several strongly divergent lineages as *Heremites* sp., with estimated divergences of ca 5.8–8.5 Mya. In our reanalysis of cyt*b*, these Iranian lineages are closely related to, but distinct from the less divergent lineage represented by populations from Iraq and western Iran and extending into Armenia and Turkey. Further research should clarify the taxonomy of available names, particularly “*transcaucasicus*” (see Masroor et al. 2021). A comparable pattern of pronounced mitochondrial structure is also evident within *H. vittatus* (Baier et al. 2017), further supplemented by Iranian material in Bahmani et al. (2018), suggesting that this taxon may also harbour underestimated diversity requiring targeted evaluation.

In contrast, mitochondrial variation within *H. auratus* appears comparatively low (Fig. 1A, Suppl. material 1: fig. S1), and available phylogenetic evidence consistently places *H. auratus* close to the *H. septemtaeniatus* complex, consistent with their historical treatment as a single species (e.g., Khalaf 1959; Faizi and Rastegar-Pouyani 2006). Taken together, these patterns suggest uneven diversification across the genus, with extensive lineage splitting within the *H. septemtaeniatus* and *H. vittatus* complexes versus shallow structure in *H. auratus*. This contrast is compatible with a scenario in which topographic complexity and associated habitat discontinuities in Anatolia and the Levant, particularly the Anatolian Highlands, have promoted regional divergence in some lineages, whereas others may reflect more recent range expansion and/or higher levels of gene flow.

Within the same conceptual framework, the deep divergence and geographic isolation of *H. afghanicus* sp. nov. seem consistent with expected but only indirectly studied mountain-driven speciation in the Hindu Kush, where long-term fragmentation along elevational and valley systems could have promoted lineage persistence and differentiation of small vertebrates (Agarwal et al. 2014, 2022; Gowande et al. 2021; Hofmann et al. 2023). Two scenarios remain plausible: the species may represent a relic of a historically wider distribution subsequently fragmented by aridification and the development of unsuitable lowland barriers (see Jablonski et al. 2024), or it may have originated through *in situ* divergence in the Hindu Kush foothills under long-term topographic and ecological isolation. Discriminating between these alternatives will require further genomic sampling across the putative gap regions and model-based biogeographic inference that incorporates past habitat suitability and range dynamics. Thus, further research in the genus *Heremites* should focus on genetic and distributional limits and address questions related to contact zones, lineage boundaries, and potential cryptic diversity across North Africa, Anatolia, the Levant, Iran, and the southern Hindu Kush.

## Supplementary Material

XML Treatment for Heremites afghanicus

## Additional information

### Artificial Intelligence (AI) use

The authors accept full responsibility for the content of the manuscript, including the disclosure of any use of AI.

No AI tools were used in the preparation of this manuscript.

### Author ORCIDs

D. Jablonski https://orcid.org/0000-0002-5394-0114

S. Hofmann https://orcid.org/0000-0003-0621-9049

M. Idrees https://orcid.org/0009-0009-2902-9679

M.A. Khan https://orcid.org/0000-0003-1980-0916

Q. Jamal https://orcid.org/0000-0002-0511-0853

W. Böhme https://orcid.org/0000-0002-8387-1287

R. Masroor https://orcid.org/0000-0001-6248-546X

### Data availability

All of the data that support the findings of this study are available in the main text or Supplementary Information.

## Supplementary materials

Supplementary material 1 Supplementary tables and figures Data type pdf Explanation note **table SS1**. Sequences used for cytochrome *b* dataset and for geographic assignment of genotypes. **table SS2**. Sequences used for the concatenated phylogeny. **table S3**. Sequences used for the nuclear allele networks. The list of morphologically examined specimens in this study. **fig. S1**. The cytochrome *b* maximum-likelihood phylogeny of the genus *Heremites*. **fig. S2**. Time-calibrated phylogeny of the genus *Heremites*. https://binary.pensoft.net/file/1765032This dataset is made available under the Open Database License (http://opendatacommons.org/licenses/odbl/1.0/). The Open Database License (ODbL) is a license agreement intended to allow users to freely share, modify, and use this Dataset while maintaining this same freedom for others, provided that the original source and author(s) are credited. Daniel Jablonski, Sylvia Hofmann, Muhammad Idrees, Muazzam Ali Khan, Qaisar Jamal, Wolfgang Böhme, Rafaqat Masroor
