# Supplementary material for: A new species of Heremites Gray, 1845 (Squamata, Scincidae) from the transboundary Hindu Kush foothills
Source: Zookeys. 2026 Sep 4;1291:111–36. doi: 10.3897/zookeys.1291.1863652 (PMC13570255; doi:10.3897/zookeys.1291.1863652)
Supplement: Supplementary file 1 [file zookeys-1291-111_article-186365__-s001.pdf]

A peer-reviewed open-access journal
ZooKeys
Launched to accelerate biodiversity research

Supplementary material for

A new species of *Heremites* Gray, 1845 (Squamata: Scincidae) from the transboundary Hindu Kush foothills

Daniel Jablonski, Sylvia Hofmann, Muhammad Idrees, Muazzam Ali Khan, Qaisar Jamal, Wolfgang Böhme, Rafaqat Masroor

Supplementary data

Table S1. Sequences used for cytochrome *b* dataset (Carranza and Arnold 2003, Güçlü et al. 2014, Bahmani et al. 2018) and for geographic assignment of genotypes (Baier et al. 2017).

Accession number	species	voucher/sample number	country: locality	source
AY151493.1	*Heremites vittatus* complex	E111039	Tunisia: Tozeur	Carranza & Arnold (2003)
AY151494.1	*Heremites vittatus* complex	E111042	Turkey: Osmandere	Carranza & Arnold (2003)
AY151495.1	*Heremites vittatus* complex	E111043	Turkey: Osmandere	Carranza & Arnold (2003)
AY151507.1	*Heremites auratus*	E11102	Turkey: Kisehir	Carranza & Arnold (2003)
AY151514.1	*Heremites vittatus* complex	E111040	Tunisia: Tozeur	Carranza & Arnold (2003)
EU443142.1	*Heremites vittatus* complex	BEV:1446	Turkey: Osmandere	Güçlü et al. (2014)
KC143201.1	*Heremites vittatus* complex	1V/Hap1	Turkey: Şanlıurfa Province: Hilvan	Güçlü et al. (2014)
KC143202.1	*Heremites vittatus* complex	5V/Hap2	Turkey: Adıyaman Province: Araban	Güçlü et al. (2014)
KC143203.1	*Heremites vittatus* complex	8V/Hap3	Turkey: Batman Province: Hasankeyf	Güçlü et al. (2014)
KC143204.1	*Heremites vittatus* complex	9V/Hap4	Turkey: Mardin Province	Güçlü et al. (2014)
KC143205.1	*Heremites vittatus* complex	14V/Hap5	Turkey: Mersin Province: Aydıncık	Güçlü et al. (2014)
KC143206.1	*Heremites vittatus* complex	28V/Hap6	Turkey: Adıyaman Province: Kahta	Güçlü et al. (2014)
KC143207.1	*Heremites vittatus* complex	29V/Hap7	Turkey: Kilis Province: Kemaliye	Güçlü et al. (2014)
KC143208.1	*Heremites vittatus* complex	23V/Hap8	Turkey: Hatay Province: Belen	Güçlü et al. (2014)
KC143209.1	*Heremites vittatus* complex	26V/Hap9	Turkey: Şanlıurfa Province: Ceylanpınar	Güçlü et al. (2014)
KC143210.1	*Heremites vittatus* complex	28AV/Hap10	Turkey: Şanlıurfa Province: Ceylanpınar	Güçlü et al. (2014)
KC143211.1	*Heremites vittatus* complex	43V/Hap11	Turkey: Siirt Province: Kurtalan	Güçlü et al. (2014)
KC143212.1	*Heremites vittatus* complex	4V/Hap12	Turkey: Diyarbakır Province: Siverek	Güçlü et al. (2014)
KC143213.1	*Heremites vittatus* complex	15V/Hap13	Turkey: Mersin Province: Gülnar	Güçlü et al. (2014)
KC143214.1	*Heremites vittatus* complex	21V/Hap14	Turkey: Gaziantep Province: Burç	Güçlü et al. (2014)
KC143215.1	*Heremites vittatus* complex	25V/Hap15	Turkey: Kilis Province: Polateli	Güçlü et al. (2014)
KC143216.1	*Heremites vittatus* complex	30V/Hap16	Turkey: Adıyaman Province: Bağpınar	Güçlü et al. (2014)
KC143217.1	*Heremites vittatus* complex	37V/Hap17	Turkey: Mardin Province: Kızıltepe	Güçlü et al. (2014)
KC143218.1	*Heremites auratus*	1A/Hap18	Turkey: Muğla Province: Dalyan	Güçlü et al. (2014)
KC143219.1	*Heremites auratus*	10A/Hap19	Turkey: Konya Province: Meke	Güçlü et al. (2014)
KC143220.1	*Heremites septemtaeniatus* complex	13A/Hap20	Turkey: Şanlıurfa Province: Harran	Güçlü et al. (2014)
KC143221.1	*Heremites auratus*	21A/Hap21	Turkey: Muğla Province: Köyceğiz	Güçlü et al. (2014)
KC143222.1	*Heremites auratus*	24A/Hap22	Turkey: Şanlıurfa Province: Viranşehir	Güçlü et al. (2014)
KC143223.1	*Heremites auratus*	25A/Hap23	Turkey: Şanlıurfa Province: Halfeti	Güçlü et al. (2014)
KC143224.1	*Heremites auratus*	26A/Hap24	Turkey: Şanlıurfa Province: Ceylanpınar	Güçlü et al. (2014)
KC143225.1	*Heremites auratus*	27A/Hap25	Turkey: Şanlıurfa Province: Birecik	Güçlü et al. (2014)
KC143226.1	*Heremites auratus*	29A/Hap26	Turkey: Batman Province: Hasankeyf	Güçlü et al. (2014)
KC143227.1	*Heremites auratus*	30A/Hap27	Turkey: Kilis Province: Yavuzeli	Güçlü et al. (2014)
KC143228.1	*Heremites auratus*	31A/Hap28	Turkey: Şırnak Province: Cizre	Güçlü et al. (2014)
KC143229.1	*Heremites auratus*	33A/Hap29	Turkey: Şanlıurfa Province: Halfeti	Güçlü et al. (2014)
KC143230.1	*Heremites auratus*	34A/Hap30	Turkey: Şanlıurfa Province: Ceylanpınar	Güçlü et al. (2014)

KC143231.1	*Heremites auratus*	35A/Hap31	Turkey: Şırnak Province: Cizre	Güçlü et al. (2014)
KC143232.1	*Heremites auratus*	36A/Hap32	Turkey: Kilis Province: Acar köyü	Güçlü et al. (2014)
KC143233.1	*Heremites auratus*	39A/Hap33	Turkey: Şanlıurfa Province: Bozova	Güçlü et al. (2014)
KC143234.1	*Heremites auratus*	40A/Hap34	Turkey: Gaziantep Province: Dokurcun	Güçlü et al. (2014)
KC143235.1	*Heremites auratus*	41A/Hap35	Turkey: Şanlıurfa Province: Küçükalanlı	Güçlü et al. (2014)
KC143236.1	*Heremites auratus*	42A/Hap36	Turkey: Muğla Province: Marmaris	Güçlü et al. (2014)
KC143237.1	*Heremites auratus*	45A/Hap37	Turkey: Diyarbakır Province: Ergani	Güçlü et al. (2014)
KC143238.1	*Heremites auratus*	47A/Hap38	Turkey: Aydın Province: Çine	Güçlü et al. (2014)
KC171165.1	*Heremites auratus*	23A/Hap19	Turkey: Konya Province: Meke	Güçlü et al. (2014)
KC171166.1	*Heremites auratus*	48A/Hap38	Turkey: Aydın Province: Çine	Güçlü et al. (2014)
MF101923.1	*Heremites septemtaeniatus* complex	IPMB47606	Iran: 47km north of Kermanshah	Baier et al. (2017)
MF101924.1	*Heremites septemtaeniatus* complex	IPMB47609	Iran: Kurdestan province: Sarvabad	Baier et al. (2017)
MF101925.1	*Heremites septemtaeniatus* complex	IPMB47605	Iran: Northwest of Khorasan around Jajarm town	Baier et al. (2017)
MF101926.1	*Heremites septemtaeniatus* complex	ZFMK 84085	Iran: Azarbaian: Kurh-e-Sahaud	Baier et al. (2017)
MF101927.1	*Heremites septemtaeniatus* complex	IPMB47608	Azerbaijan: West of Azerbaijan	Baier et al. (2017)
MF101928.1	*Heremites vittatus* complex	NMW 19811:3	Turkey: 68km east of Eskisehir	Baier et al. (2017)
MF101929.1	*Heremites vittatus* complex	NMW 19811:2	Turkey: 68km east of Eskisehir	Baier et al. (2017)
MF101930.1	*Heremites vittatus* complex	NMW 19811:1	Turkey: Southwest of Eskisehir	Baier et al. (2017)
MF101931.1	*Heremites vittatus* complex	BEV 1514	Turkey: 2.5km northeast of Olukkoyagi (Southwest of Pozanti)	Baier et al. (2017)
MF101932.1	*Heremites vittatus* complex	IPMB47604	Iran: 160km west of Kermanshah around Ghasr-Shirin town	Baier et al. (2017)
MF101933.1	*Heremites vittatus* complex	IPMB47603	Iran: 160km west of Kermanshah around Ghasr-Shirin town	Baier et al. (2017)
MF101934.1	*Heremites vittatus* complex	IPMB47602	Iran: 120km west of Kermanshah Rijab area	Baier et al. (2017)
MF101935.1	*Heremites vittatus* complex	NHMC 80.3.81.5	Syria: 40km south of Homs	Baier et al. (2017)
MF101936.1	*Heremites vittatus* complex	NHMC 80.3.81.16	Syria: 40km east of Homs	Baier et al. (2017)
MF101937.1	*Heremites vittatus* complex	NHMC 80.3.81.15	Syria: 40km east of Homs	Baier et al. (2017)
MF101938.1	*Heremites vittatus* complex	NHMC 80.3.81.4	Syria: Buhayrat Qattinah, lake near Homs	Baier et al. (2017)
MF101939.1	*Heremites vittatus* complex	NHMC 80.3.81.33	Syria: Krak des Chevaliers	Baier et al. (2017)
MF101940.1	*Heremites vittatus* complex	NHMC 80.3.81.31	Syria: Al Hamidiyah	Baier et al. (2017)
MF101941.1	*Heremites vittatus* complex	NHMC 80.3.81.30	Syria: Al Hamidiyah	Baier et al. (2017)
MF101942.1	*Heremites vittatus* complex	NHMC 80.3.81.40	Syria: Maquam Assayedh 20km north Lattakia	Baier et al. (2017)
MF101943.1	*Heremites vittatus* complex	NHMC 80.3.81.39	Syria: Maquam Assayedh 20km north Lattakia	Baier et al. (2017)
MF101944.1	*Heremites vittatus* complex	NHMC 80.3.81.7	Syria: Slinbah, 60km east of Lattakia: 1200m alt.	Baier et al. (2017)
MF101945.1	*Heremites vittatus* complex	NHMC 80.3.81.20	Syria: Alawit Mountain range, Al Haffah, 40km east of Lattakia: 600m alt.	Baier et al. (2017)
MF101946.1	*Heremites vittatus* complex	NHMC 80.3.81.19	Syria: Alawit Mountain range, Al Haffah, 40km east of Lattakia: 600m alt.	Baier et al. (2017)

MF101947.1	*Heremites vittatus* complex	NHMC 80.3.81.42	Syria: Aleppo-Lattakia, 90-95km east of Lattakia, 2-3km after Ariha	Baier et al. (2017)
MF101948.1	*Heremites vittatus* complex	NHMC 80.3.81.12	Syria: just before Kanateh Tabtanaz village, 37km southwest of Aleppo	Baier et al. (2017)
MF101949.1	*Heremites vittatus* complex	NHMC 80.3.81.2	Syria: Nizran, 25km south of As Suwayda	Baier et al. (2017)
MF101950.1	*Heremites vittatus* complex	ZFMK 64971	Lebanon: Djebel Barouk (east slope) above Kefraiya	Baier et al. (2017)
MF101951.1	*Heremites vittatus* complex	ZFMK 65003	Lebanon: Mt. Lebanon: above Bcharré	Baier et al. (2017)
MF101952.1	*Heremites vittatus* complex	ZFMK 60995	Lebanon: Mt. Lebanon: East of Bcharré	Baier et al. (2017)
MF101953.1	*Heremites vittatus* complex	ZFMK 65011	Lebanon: Mt. Lebanon: cedars of Tannourine	Baier et al. (2017)
MF101954.1	*Heremites vittatus* complex	HUJR-23674	Israel: Bet Guvrin area: Horvat 'Abia	Baier et al. (2017)
MF101955.1	*Heremites vittatus* complex	HUJR-23675	Israel: Bet Guvrin area: Horvat 'Abia	Baier et al. (2017)
MF101956.1	*Heremites vittatus* complex	HUJR-23677	Israel: Bet Guvrin area: Horvat 'Abia	Baier et al. (2017)
MF101957.1	*Heremites vittatus* complex	HUJR-23678	Israel: Bet Guvrin area: Horvat 'Abia	Baier et al. (2017)
MF101958.1	*Heremites vittatus* complex	NHMC 80.3.81.53	Jordan: North Shuna	Baier et al. (2017)
MF101959.1	*Heremites vittatus* complex	NHMC 80.3.81.54	Jordan: North Shuna	Baier et al. (2017)
MF101960.1	*Heremites vittatus* complex	NHMC 80.3.81.43	Jordan: Al Himma	Baier et al. (2017)
MF101961.1	*Heremites vittatus* complex	NHMC 80.3.81.44	Jordan: Al Himma	Baier et al. (2017)
MF101962.1	*Heremites vittatus* complex	ZSM	Cyprus: Latchi, Elia Apts	Baier et al. (2017)
MF101963.1	*Heremites vittatus* complex	NHMC 80.3.81.47	Cyprus: Cape Gkrecko and Agioi Anargyroi	Baier et al. (2017)
MF101964.1	*Heremites vittatus* complex	SPM003133	Cyprus: Vrysoulles	Baier et al. (2017)
MF101965.1	*Heremites vittatus* complex	SPM003119	Cyprus: Vrysoulles	Baier et al. (2017)
MF101966.1	*Heremites vittatus* complex	ZFMK unknown	Egypt: Rashid, Guava plantation	Baier et al. (2017)
MF101967.1	*Heremites vittatus* complex	ZFMK unknown	Egypt: Rashid	Baier et al. (2017)
MF101968.1	*Heremites vittatus* complex	SPM002950(76)	Egypt: W Alexandria	Baier et al. (2017)
MF101969.1	*Heremites vittatus* complex	NHMC 80.3.81.1	Libya: Archaia Kyrrini	Baier et al. (2017)
MF101970.1	*Heremites vittatus* complex	T31-40 Mabuvita1	Tunisia: Tozeur City (Palermal)	Baier et al. (2017)
MF101971.1	*Heremites vittatus* complex	T31-36 Mabuvita2	Tunisia: 5km from Gafsa direction Tozeur	Baier et al. (2017)
MF101972.1	*Heremites vittatus* complex	T31-36 Mabuvita1	Tunisia: 5km from Gafsa direction Tozeur	Baier et al. (2017)
MF590341.1	*Heremites septemtaeniatus* complex	ERP487	Iran: Khomarlo: Khoda Afarin	Bahmani et al. (2018)
MF590342.1	*Heremites septemtaeniatus* complex	RUZMZB5634	Iran: Takab: Amin Abad Village	Bahmani et al. (2018)
MF590343.1	*Heremites septemtaeniatus* complex	RUZMZB5636	Iran: Urmia Shahi Island	Bahmani et al. (2018)
MF590344.1	*Heremites septemtaeniatus* complex	RUZMZB5637	Iran: Marakan Village	Bahmani et al. (2018)
MF590345.1	*Heremites septemtaeniatus* complex	RUZMZB5638	Iran: Marakan Village	Bahmani et al. (2018)
MF590346.1	*Heremites septemtaeniatus* complex	RUZMZB5639	Iran: on the road to Mahabad Sardasht	Bahmani et al. (2018)
MF590347.1	*Heremites septemtaeniatus* complex	RUZMZB5641	Iran: Zanjan: Halab	Bahmani et al. (2018)
MF590348.1	*Heremites septemtaeniatus* complex	RUZMZB5642	Iran: Zanjan: Halab	Bahmani et al. (2018)
MF590349.1	*Heremites septemtaeniatus* complex	RUZMZB5653	Iraq: Solimanie: Coa Maci	Bahmani et al. (2018)

MF590350.1	*Heremites septemtaeniatus* complex	RUZMZB5630	Iran: Saghez: Tamugheh Village	Bahmani et al. (2018)
MF590351.1	*Heremites septemtaeniatus* complex	RUZMZB5631	Iran: Saghez: Tamugheh Village	Bahmani et al. (2018)
MF590352.1	*Heremites septemtaeniatus* complex	RUZMZB5632	Iran: on the road to Saghez	Bahmani et al. (2018)
MF590353.1	*Heremites septemtaeniatus* complex	RUZMZB5640	Iran: Sanandaj	Bahmani et al. (2018)
MF590354.1	Heremites septemtaeniatus complex (sp3)	ERP589	Iran: Khorasan Province	Bahmani et al. (2018)
MF590355.1	*Heremites septemtaeniatus* complex (sp3)	ERP1036	Iran: Khorasan Province	Bahmani et al. (2018)
MF590356.1	*Heremites septemtaeniatus* complex (sp3)	RUZMZB5656	Iran: Khorasan Province	Bahmani et al. (2018)
MF590357.1	*Heremites septemtaeniatus* complex	RUZMZB5667	Iran: Kermanshah	Bahmani et al. (2018)
MF590358.1	*Heremites septemtaeniatus* complex	RUZMZB5822	Iran: Kermanshah: Ghasreshirin	Bahmani et al. (2018)
MF590359.1	*Heremites septemtaeniatus* complex	RUZMZB5670	Iran: Hamadan: Fash Village	Bahmani et al. (2018)
MF590360.1	*Heremites septemtaeniatus* complex (sp2)	RUZMZB5643	Iran: Khuzestan Province	Bahmani et al. (2018)
MF590361.1	*Heremites septemtaeniatus* complex (sp2)	RUZMZB5823	Iran: Khuzestan Province	Bahmani et al. (2018)
MF590362.1	*Heremites septemtaeniatus* complex (sp2)	RUZMZB5644	Iran: Khuzestan Province	Bahmani et al. (2018)
MF590363.1	*Heremites septemtaeniatus* complex	RUZMZB6151	Iran: Khuzestan-Mahshahr	Bahmani et al. (2018)
MF590364.1	*Heremites septemtaeniatus* complex	RUZMZB6149	Iran: Khuzestan-Mahshahr	Bahmani et al. (2018)
MF590365.1	*Heremites septemtaeniatus* complex	RUZMZB6154	Iran: Khuzestan-Mahshahr	Bahmani et al. (2018)
MF590366.1	*Heremites septemtaeniatus* complex (sp1)	ERP1212	Iran: Bushehr Province	Bahmani et al. (2018)
MF590367.1	*Heremites septemtaeniatus* complex (sp3)	RUZMZB5657	Iran: Isfahan Province	Bahmani et al. (2018)
MF590368.1	*Heremites septemtaeniatus* complex (sp3)	RUZMZB5645	Iran: Isfahan Province	Bahmani et al. (2018)
MF590369.1	*Heremites septemtaeniatus* complex (sp3)	RUZMZB5646	Iran: Isfahan Province	Bahmani et al. (2018)
MF590370.1	*Heremites septemtaeniatus* complex (sp3)	ZUTC6147	Iran: Isfahan Province	Bahmani et al. (2018)
MF590371.1	*Heremites septemtaeniatus* complex (sp3)	ZUTC6150	Iran: Isfahan Province	Bahmani et al. (2018)
MF590372.1	*Heremites septemtaeniatus* complex	RUZMZB6035	Iran: Poldokhtar	Bahmani et al. (2018)
MF590373.1	*Heremites septemtaeniatus* complex	RUZMZB6036	Iran: Poldokhtar	Bahmani et al. (2018)
MF590374.1	*Heremites septemtaeniatus* complex	RUZMZB6037	Iran: Poldokhtar	Bahmani et al. (2018)
MF590375.1	*Heremites septemtaeniatus* complex	RUZMZB6038	Iran: Poldokhtar	Bahmani et al. (2018)
MF590376.1	*Heremites septemtaeniatus* complex (sp1)	ERP1434	Iran: Fars Province	Bahmani et al. (2018)
MF590377.1	*Heremites septemtaeniatus* complex (sp1)	ERP1562	Iran: Fars Province	Bahmani et al. (2018)
MF590378.1	*Heremites septemtaeniatus* complex (sp1)	ERP1563	Iran: Fars Province	Bahmani et al. (2018)
MF590379.1	*Heremites septemtaeniatus* complex (sp1)	RUZMZB5648	Iran: Fars Province	Bahmani et al. (2018)
MF590380.1	*Heremites septemtaeniatus* complex (sp1)	RUZMZB5649	Iran: Fars Province	Bahmani et al. (2018)
MF590381.1	*Heremites septemtaeniatus* complex (sp1)	RUZMZB5650	Iran: Fars Province	Bahmani et al. (2018)
MF590382.1	*Heremites septemtaeniatus* complex (sp1)	RUZMZB5651	Iran: Fars Province	Bahmani et al. (2018)

MF590383.1	*Heremites septemtaeniatus* complex (sp1)	RUZMZB5652	Iran: Fars Province	Bahmani et al. (2018)
MF590384.1	*Heremites septemtaeniatus* complex	RUZMZB5672	Iraq: Khaganvillage	Bahmani et al. (2018)
MF590385.1	*Heremites septemtaeniatus* complex	RUZMZB5673	Iraq: Al-Mutanazah	Bahmani et al. (2018)
MF590386.1	*Heremites septemtaeniatus* complex	RUZMZB5674	Iraq: Al-Shamiya	Bahmani et al. (2018)
MF590387.1	*Heremites septemtaeniatus* complex	RUZMZB5675	Iraq: Hilla city	Bahmani et al. (2018)
MF590388.1	*Heremites septemtaeniatus* complex	RUZMZB5677	Iraq: Khaganvillage	Bahmani et al. (2018)
MF590389.1	*Heremites septemtaeniatus* complex	RUZMZB5678	Iraq: Hillacity	Bahmani et al. (2018)
MF590390.1	*Heremites septemtaeniatus* complex	RUZMZB5679	Iraq: Al-Diwaniya	Bahmani et al. (2018)
MF590391.1	*Heremites septemtaeniatus* complex	RUZMZB5682	Iraq: Al-Shamiya	Bahmani et al. (2018)
MF590392.1	*Heremites septemtaeniatus* complex	RUZMZB5683	Iraq: Madhlum village	Bahmani et al. (2018)
MF590393.1	*Heremites septemtaeniatus* complex	RUZMZB5684	Iraq: Al-Salman	Bahmani et al. (2018)
MF590394.1	*Heremites septemtaeniatus* complex	RUZMZB5686	Iraq: Al-Shamiya	Bahmani et al. (2018)
MF590395.1	*Heremites septemtaeniatus* complex	RUZMZB5687	Iraq: Kutelgwam	Bahmani et al. (2018)
MF590396.1	*Heremites septemtaeniatus* complex	RUZMZB5688	Iraq: Al-Salman	Bahmani et al. (2018)
MF590397.1	*Heremites septemtaeniatus* complex	RUZMZB5689	Iraq: Khagan village	Bahmani et al. (2018)
MF590398.1	*Heremites septemtaeniatus* complex	RUZMZB5691	Iraq: Al-Mutanazah	Bahmani et al. (2018)
MF590399.1	*Heremites septemtaeniatus* complex	RUZMZB5692	Iraq: Al Jayir village	Bahmani et al. (2018)
MF590400.1	*Heremites septemtaeniatus* complex	RUZMZB5694	Iraq: Al Jayir village	Bahmani et al. (2018)
MF590401.1	*Heremites septemtaeniatus* complex	RUZMZB5695	Iraq: Al-Nikhealah	Bahmani et al. (2018)
MF590402.1	*Heremites septemtaeniatus* complex	RUZMZB5696	Iraq: Al-Mutanazah	Bahmani et al. (2018)
MF590403.1	*Heremites septemtaeniatus* complex	RUZMZB5697	Iraq: Madhlum village	Bahmani et al. (2018)
MF590404.1	*Heremites septemtaeniatus* complex	RUZMZB5701	Iraq: Madhlum village	Bahmani et al. (2018)
MF590405.1	*Heremites septemtaeniatus* complex	RUZMZB5704	Iraq: AL-Bathaa	Bahmani et al. (2018)
MF590406.1	*Heremites septemtaeniatus* complex	5705	Iraq: Al-Jubaila	Bahmani et al. (2018)
MF590407.1	*Heremites septemtaeniatus* complex	RUZMZB5706	Iraq: Al-Jubaila	Bahmani et al. (2018)
MF590408.1	*Heremites septemtaeniatus* complex	RUZMZB5709	Iraq: Al-Shamiya	Bahmani et al. (2018)
MF590409.1	*Heremites septemtaeniatus* complex	RUZMZB5710	Iraq: Hillacity	Bahmani et al. (2018)
MF590410.1	*Heremites septemtaeniatus* complex	RUZMZB5711	Iraq: Kutelgwam	Bahmani et al. (2018)
MF590411.1	*Heremites septemtaeniatus* complex	RUZMZB5712	Iraq: Al-Abbasiya	Bahmani et al. (2018)
MF590412.1	*Heremites septemtaeniatus* complex	RUZMZB5714	Iraq: Al-Jubaila	Bahmani et al. (2018)
MF590413.1	*Heremites vittatus* complex	RUZMZB5731	Iran: Kermanshah: Sarpol-e Zahab	Bahmani et al. (2018)
MF590414.1	*Heremites vittatus* complex	RUZMZB5734	Iran: Kermanshah: Sarpol-e Zahab	Bahmani et al. (2018)
MF590415.1	*Heremites vittatus* complex	RUZMZB5740	Iran: Kermanshah: Sarpol-e Zahab	Bahmani et al. (2018)

MF590416.1	*Heremites vittatus* complex	RUZMZB5727	Iran: Kermanshah: Sarpol-e Zahab	Bahmani et al. (2018)
MF590417.1	*Heremites vittatus* complex	RUZMZB5733	Iran: Ilam: Darreh Shahr	Bahmani et al. (2018)
MF590418.1	*Heremites vittatus* complex	RUZMZB5723	Iran: Ilam: Darreh Shahr	Bahmani et al. (2018)
MF590419.1	*Heremites vittatus* complex	RUZMZB5724	Iran: Ilam: Darreh Shahr	Bahmani et al. (2018)
MF590420.1	*Heremites vittatus* complex	RUZMZB5729	Iran: Lorestan: Pol-e-Dokhtar	Bahmani et al. (2018)
MF590421.1	*Heremites vittatus* complex	RUZMZB5732	Iran: Lorestan: Pol-e-Dokhtar	Bahmani et al. (2018)
MF590422.1	*Heremites vittatus* complex	RUZMZB5739	Iran: Lorestan: Pol-e-Dokhtar	Bahmani et al. (2018)
MF590423.1	*Heremites vittatus* complex	RUZMZB5715	Iraq: Khagan village	Bahmani et al. (2018)
MF590424.1	*Heremites vittatus* complex	RUZMZB5716	Iraq: Khagan village	Bahmani et al. (2018)
MF590425.1	*Heremites vittatus* complex	RUZMZB5717	Iraq: Khagan village	Bahmani et al. (2018)
MF590426.1	*Heremites vittatus* complex	RUZMZB5722	Iran: Ilam: Darreh Shahr	Bahmani et al. (2018)
PZ334412	*Heremites septemtaeniatus* complex	2917	Armenia: Aruch	This study
PZ334413	*Heremites septemtaeniatus* complex	2922	Armenia: Aruch	This study
PZ334414	*Heremites vittatus* complex	6735	Lebanon: Daraiya	This study
PZ334415	*Heremites vittatus* complex	6777	Lebanon: Houch El Saalouk	This study
PZ334416	*Heremites vittatus* complex	6792	Lebanon: Aayoun El Siman	This study
PZ334417	*Heremites vittatus* complex	6803	Lebanon: Faqra	This study
PZ334418	*Heremites vittatus* complex	8139	Cyprus	This study
PZ334419	*Heremites vittatus* complex	8595	Iraq: Shaqlawa	This study
PZ334420	*Heremites vittatus* complex	8607	Iraq: Banoka	This study
PZ334421	*Heremites vittatus* complex	8610	Iraq: Banatan	This study
PZ334422	*Heremites vittatus* complex	8639	Iraq: Sulaymaniyah	This study
PZ334423	*Heremites septemtaeniatus* complex	8655	Iraq: Darbandikhan	This study
PZ334424	*Heremites afghanicus* sp. nov.	NHMW-Zoo-HS-42309-1	Pakistan: Shah Alam Baba	This study

Table S2. Sequences used for the concatenated phylogeny.

Species	ID	12S	16S	Cyt *b*	ND2	MC1R	RAG1	Source
Ablepharus himalayanus	mitogenome	MN885892	MN885892	MN885892	MN885892	-	-	Weng (unpublished)
Asymblepharus sikimmensis	mitogenome	PP439832	PP439832	PP439832	PP439832	-	-	Weng and Peng (unpublished)
Caledoniscincus austrocaledonicus	EUG3	EU567931	EU567922	EU567835	EU567727		EU568024	Chapple et al. (2009)
Caledoniscincus austrocaledonicus	AMSR163262	-	KX231459	-	KX231474	KX231407	KX231389	Karin et al. (2016)
Cordylus angolensis	NB 0612	OQ869867	OQ869891	-	OQ869522	-	-	Bates et al. (2023)
Cordylus minor	PEMR 16376	HQ167059	HQ167170	-	HQ166959	JQ389816	-	Greenbaum et al. (2012), Stanley et al. (2011)
Cordylus beraduccii	WRB 0037	HQ167061	HQ167172	-	KT941395	JQ389825	-	Greenbaum et al. (2012), Stanley et al. (2011, 2016)
Cryptoblepharus novocaledonicus	AMSR163245	-	KX231460	-	KX231475	KX231408	KX231390	Karin et al. (2016)
Dasia olivacea	BRK 392	-	KX231445	-	KX231463	KX231393	KX231375	Karin et al. (2016)
Dasia vittata	BRK 391	-	KX231446	-	KX231464	KX231394	KX231376	Karin et al. (2016)
Eumeces algeriensis algeriensis	E1009.4	EU278017	EU278082	EU278249	-	-	-	Carranza et al. (2008)
Eumeces algeriensis algeriensis	E1009.5	EU278018	EU278083	EU278250	-	-	-	Carranza et al. (2008)
Eumeces schneideri aldrovandii	E1009.6	EU278005	EU278072	EU278237	-	-	-	Carranza et al. (2008)
Eumeces schneideri aldrovandii	E1009.7	EU278004	EU278071	EU278236	-	-	-	Carranza et al. (2008)
Eumecia anchietae	PEMR 16786	-	KX231447	-	-	KX231395	KX231377	Karin et al. (2016)
Eumecia anchietae	PEMR 16779	-	KX231448	-	-	KX231396	KX231378	Karin et al. (2016)
Eurylepis taeniolatus	MVZ 250452	JQ344281	JQ344270	JQ344292	-	-	JQ344256	Lavin and Papenfuss (2012)
Eutropis trivittata	MVZ 248450	-	KX364958	-	KX365037	KX365017	KX365045	Karin et al. (2016)
Eutropis indeprensa	FMNH 282851	-	KX364954	-	KX365033	KX365013	KX365041	Karin et al. (2016)
Eutropis longicaudata	MZKUR 00704, FMNH 255526	-	KX231449	DQ239139	KX231465	KX231397	KX231379	Whiting et al. (2006), Karin et al. (2016)
Eutropis macularia	CAS 247949, FMNH 258873	-	KX231450	DQ239136	KX231466	KX231398	KX231380	Whiting et al. (2006), Karin et al. (2016)
Eutropis multifasciata	HZF 306	ON746667	ON746667	ON746667	ON746667	-	-	Jahari et al. (unpublished)
Eutropis multifasciata	FMNH 269170, FMNH 255530	-	KX231451	DQ239138	KX231467	KX231399	KX231381	Whiting et al. (2006), Karin et al. (2016)
Eutropis rudis	FMNH 239732	-	KX231452	DQ239135	KX231468	KX231400	KX231382	Whiting et al. (2006), Karin et al. (2016)
Eutropis rugifera	FMNH 273652	-	KX364957	-	KX365036	KX365016	KX365044	Karin et al. (2016)
Heremites afghanicus sp. nov.	NHMW-Zoo-HS-42309-1	PZ348441	PZ348454	PZ334424	PZ338262	PZ661696	PZ661709	This study
Heremites auratus	ZFMK 75837	-	KX364961	-	GU931545	-	KX365048	Portik et al. (2010), Karin et al. (2016)
Heremites septemtaeniatus complex	2917	PZ348429	PZ348442	PZ334412	-	PZ661690	PZ661698	This study
Heremites septemtaeniatus complex	2922	PZ348430	PZ348443	PZ334413	-	PZ661691	-	This study
Heremites septemtaeniatus complex	8655	PZ348440	PZ348453	PZ334423	-	PZ661697	PZ661707	This study
Heremites septemtaeniatus	ZFMK 84085	-	KX364964	-	GU931546	KX365022	KX365050	Portik et al. (2010), Karin et al. (2016)
Heremites vittatus complex	6735	PZ348431	PZ348444	PZ334414	PZ338253	-	PZ661699	This study
Heremites vittatus complex	6777	PZ348432	PZ348445	PZ334415	PZ338254	PZ661692	PZ661700	This study
Heremites vittatus complex	6792	PZ348433	PZ348446	PZ334416	PZ338255	PZ661693	PZ661701	This study
Heremites vittatus complex	6803	PZ348434	PZ348447	PZ334417	PZ338256	-	PZ661702	This study
Heremites vittatus complex	8139	PZ348435	PZ348448	PZ334418	PZ338257	-	PZ661703	This study
Heremites vittatus complex	8595	PZ348436	PZ348449	PZ334419	PZ338258	-	PZ661704	This study
Heremites vittatus complex	8607	PZ348437	PZ348450	PZ334420	PZ338259	-	PZ661705	This study
Heremites vittatus complex	8610	PZ348438	PZ348451	PZ334421	PZ338260	PZ661694	PZ661706	This study
Heremites vittatus complex	8639	PZ348439	PZ348452	PZ334422	PZ338261	PZ661695	PZ661708	This study
Heremites vittatus complex	E111039	AY151421	AY151455	AY151493	-	-	-	Carranza and Arnold (2003)

Heremites vittatus complex	E111042	AY151422	AY151456	AY151494	-	-	-	Carranza and Arnold (2003)
Heremites vittatus complex	E111043	AY151423	AY151457	AY151495	-	-	-	Carranza and Arnold (2003)
Heremites vittatus complex	E111040	AY151442	AY151488	AY151514	-	-	-	Carranza and Arnold (2003)
Chioninia delalandii	BMNH 2000.18.M45	-	AY151482	-	KX231461	KX231391	KX231373	Carranza and Arnold (2003), Karin et al. (2016)
Chioninia vaillantii	BMNH 2000.10.M50	-	-	-	KX231462	KX231392	-	Karin et al. (2016)
Marisora aurulae	CAS 231775, USNM 268428	-	KX364959	JN246081	-	KX365018	KX365046	Karin et al. (2016)
Namazonurus namaquensis	C4	AY218000	AY217950	EU116507	-	-	-	Whiting et al. (2003), Noonan et al. (2013)
Notomabuya frenata	UAM 60, SC 28	-	KX231453	DQ239173	KX231469	KX231401	KX231383	Whiting et al. (2006), Karin et al. (2016)
Manciola guaporicola	UAM 104, LG 1574	-	KX364960	DQ239169	KX365038	KX365019	KX365047	Whiting et al. (2006), Karin et al. (2016)
Ninurta coeruleopunctatus	KTH 329	HQ167067	HQ167178	-	HQ166966	-	-	Karin et al. (2016)
Toenayar novemcarinata	CAS 213820	-	KX364955	-	KX365034	KX365014	KX365042	Karin et al. (2016)
Toenayar novemcarinata	CAS 216022	-	KX364956	-	KX365035	KX365015	KX365043	Karin et al. (2016)
Trachylepis auropunctata	PEM Field No 437	-	KX364962	MK792080	KX365039	KX365020	KX365049	Karin et al. (2016), Weinell et al. (2019)
Trachylepis boulengeri	PEMR 5533	-	KX231454	MK792081	KX231470	KX231402	KX231384	Karin et al. (2016), Weinell et al. (2019)
Trachylepis depressa	PEMR 17745, PEMR 15573	-	KX231455	MK792086	-	KX231403	KX231385	Karin et al. (2016), Weinell et al. (2019)
Trachylepis paucisquamis	UWBM 6041, PEM R4436	-	KX364963	MK792102	KX365040	KX365021	-	Karin et al. (2016), Weinell et al. (2019)

Table S3. Sequences used for the allele nuclear networks.

Species	ID	MC1R	RAG1	Source
Heremites afghanicus sp. nov.	NHMW-Zoo-HS-42309-1	PZ661696	PZ661709	This study
Heremites auratus	ZFMK 75837	-	KX365048	Karin et al. (2016)
Heremites septemtaeniatus	CAS 179697	-	AY662629	Townsend et al. (2004)
Heremites septemtaeniatus	ZFMK 84085	KX365022	KX365050	Karin et al. (2016)
Heremites septemtaeniatus	2917	PZ661690	PZ661698	This study
Heremites septemtaeniatus	2922	PZ661691	-	This study
Heremites septemtaeniatus	8655	PZ661697	PZ661707	This study
Heremites vittatus	6735	-	PZ661699	This study
Heremites vittatus	6777	PZ661692	PZ661700	This study
Heremites vittatus	6792	PZ661693	PZ661701	This study
Heremites vittatus	6803	-	PZ661702	This study
Heremites vittatus	8139	-	PZ661703	This study
Heremites vittatus	8595	-	PZ661704	This study
Heremites vittatus	8607	-	PZ661705	This study
Heremites vittatus	8610	PZ661694	PZ661706	This study
Heremites vittatus	8639	PZ661695	PZ661708	This study

Supplementary text 1. The list of morphologically examined specimens in this study.

Heremites afghanicus **sp. nov.**, Afghanistan: Nangarhar Province: Dar-e-Nur, vic. Shewa: **ZFMK 9064**
Heremites afghanicus **sp. nov.**, Pakistan: Khyber Pakhtunkhwa: Shah Alam Baba, Lower Dir: **NHMW-Zoo-HS-42309-1 (DJ9560)**; **PMNH 3474–3478**; **PMNH 3518–3524**
Heremites auratus, Turkey: Gaziantep Province: between Ömeroglo & Sahinbey, 10 km NE of Polateli, northern side of valley: **ZFMK 56965**
Heremites auratus, Turkey: Mersin Province (İçel): Doline Kanlidivane, near Kızkalesi: **ZFMK 57086**
Heremites auratus, Turkey: Mersin Province: north of Mersin, Gözne Köy: **ZFMK 68537**
Heremites auratus, Turkey: Şanlıurfa Province (Urfa): Euphrates Valley, ca. 3 km N of Halfeti: **ZFMK 75837**
Heremites auratus, Turkey: Mardin Province: Mardin Dağları, 8 km NE of Derik: **ZFMK 75863**
Heremites auratus, Turkey: Kahramanmaraş Province (Maraş): 12 km E of Pazarcık: **ZFMK 75880**
Heremites septemtaeniatus, Iran: Fars, Bushehr Peninsula: **ZFMK 26842–26844**
Heremites septemtaeniatus, Iran: Fars: NW of Shiraz, Yasuj, Kakan village: **ZFMK 71601**
Heremites septemtaeniatus, Syria: Abu Kamal: **ZFMK 71726**
Heremites septemtaeniatus, Turkey: Şanlıurfa Province: Birecik, Euphrates: **ZFMK 13939**
Heremites septemtaeniatus, Turkmenistan: 200 km W of Ashgabat: **ZFMK 53810**
Heremites vittatus, Turkey: Bayburt Province: Harmanözü: Candan et al. (2019) (n=2, unvouchered)

Figure S1. The cytochrome *b* maximum likelihood phylogeny of the genus *Heremites*.

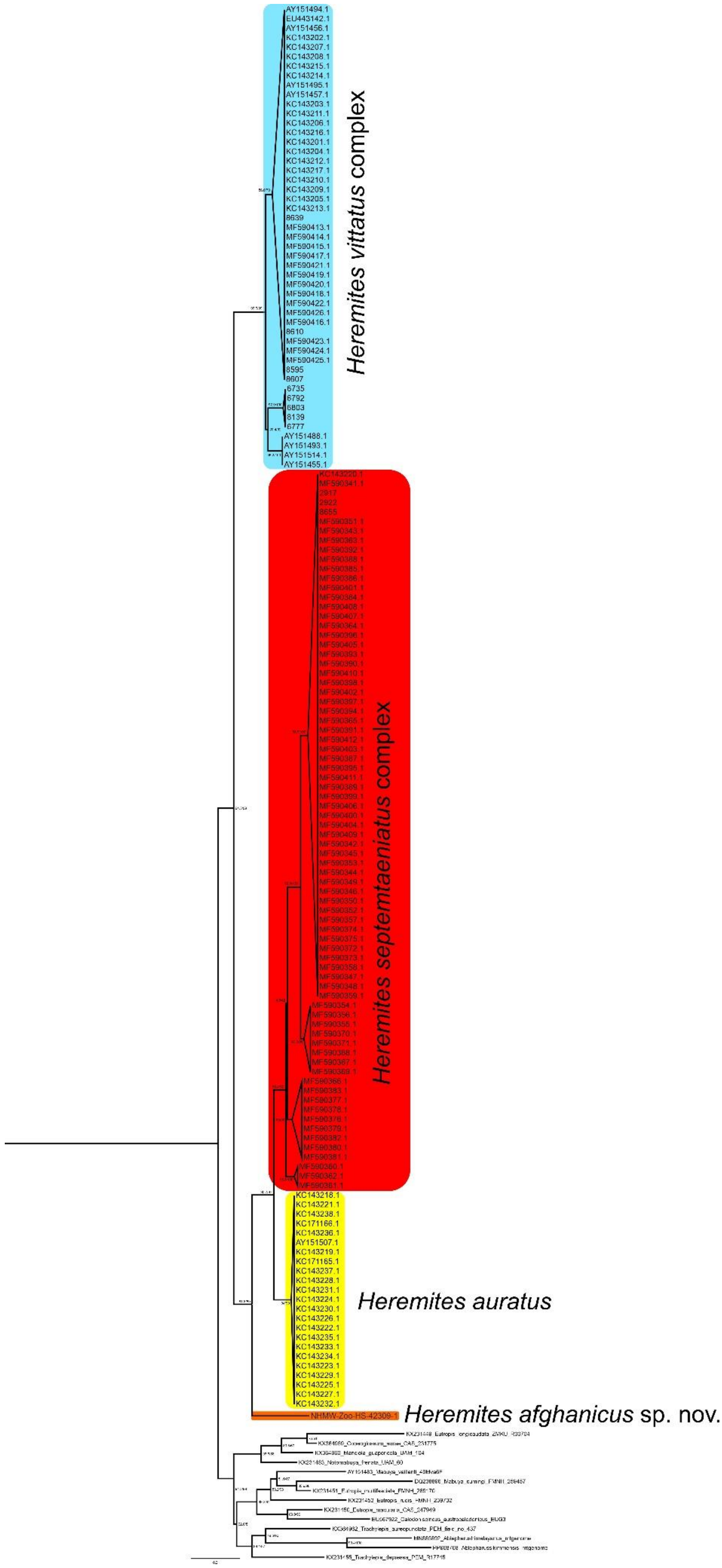

Figure S2. Time calibrated phylogeny of the genus *Heremites*. Numbers besides branches indicate mean estimated node ages (in millions of years) and, while blue bars represent the corresponding 95% highest posterior densities (HPD) intervals. Red circles indicate calibration points.

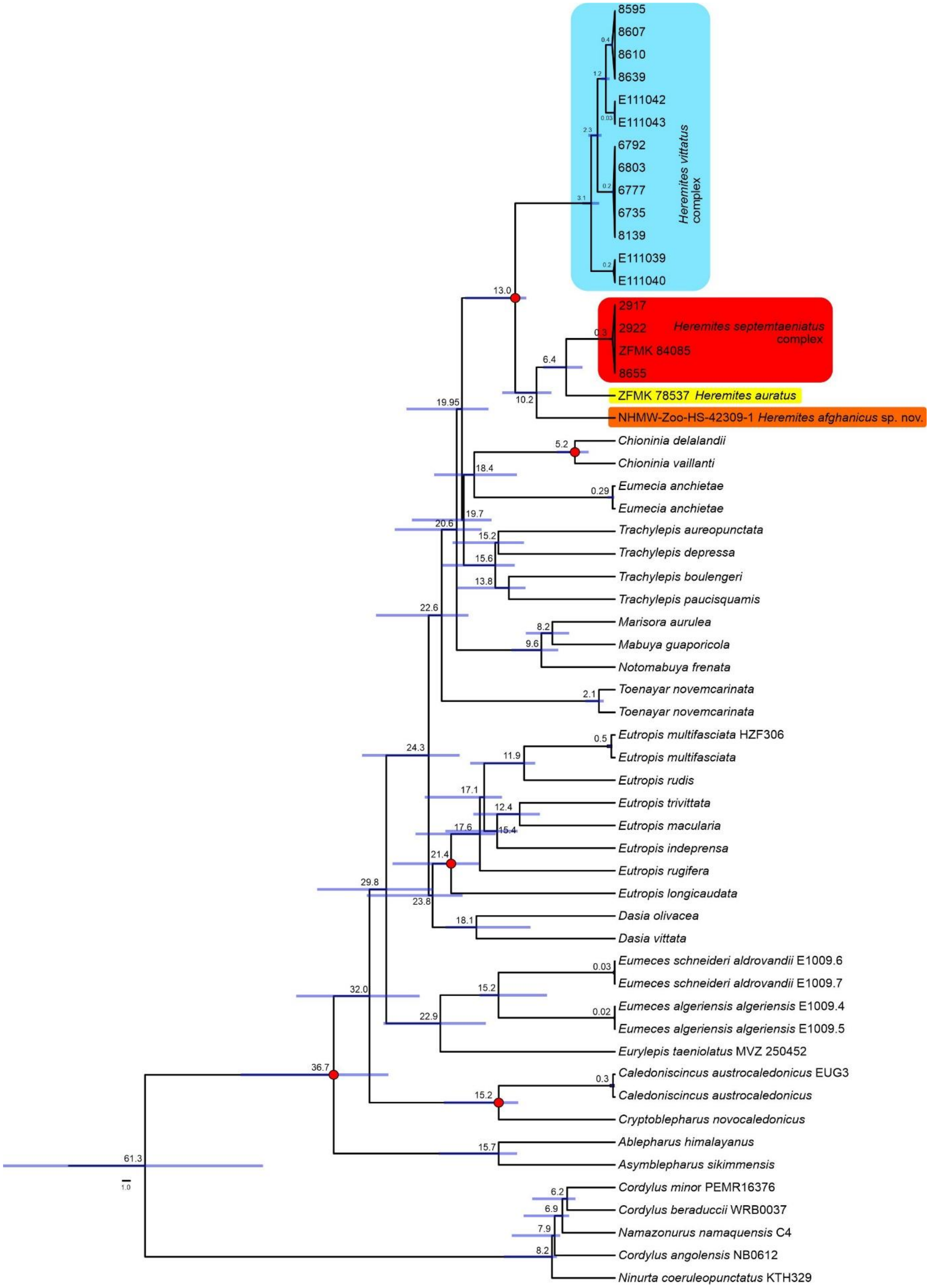

References

Bahmani Z, Rastegar-Pouyani E, Rastegar-Pouyani N (2018) The phylogenetic relationships and molecular systematics of scincid lizards of the genus *Heremites* (Sauria, Scincidae) in the Middle East based on mtDNA sequences. Mitochondrial DNA Part A 29: 846–855. https://doi.org/10.1080/24701394.2017.1373107

Baier F, Schmitz A, Sauer-Gürth H, Wink M (2017) Pre-Quaternary divergence and subsequent radiation explain longitudinal patterns of genetic and morphological variation in the striped skink, *Heremites vittatus*. BMC Evolutionary Biology 17: 132. https://doi.org/10.1186/s12862-017-0969-0

Bates MF, Lobón-Rovira J, Stanley EL, Branch WR, Vaz Pinto P (2023) A new species of green-eyed Cordylus Laurenti, 1768 from the west-central highlands of Angola, and the rediscovery of *Cordylus angolensis* (Bocage, 1895) (Squamata: Cordylidae). Vertebrate Zoology 73: 599–646. https://doi.org/10.3897/vz.73.e95639

Carranza S, Arnold EN (2003) Investigating the origin of transoceanic distributions: Mtdna shows *Mabuya* lizards (Reptilia, Scincidae) crossed the Atlantic twice. Systematics and Biodiversity 1: 275–282. http://doi.org/10.1017/S1477200003001099

Carranza S, Arnold EN, Geniez P, Roca J, Mateo JA (2008) Radiation, multiple dispersal and parallelism in the skinks, *Chalcides* and *Sphenops* (Squamata: Scincidae), with comments on *Scincus* and *Scincopus* and the age of the Sahara Desert. Molecular Phylogenetics and Evolution 46(3): 1071–1094. https://doi.org/10.1016/j.ympev.2007.11.018

Chapple DG, Ritchie PA, Daugherty CH (2009) Origin, diversification, and systematics of the New Zealand skink fauna (Reptilia: Scincidae). Molecular Phylogenetics and Evolution 52(2): 470–487. https://doi.org/10.1016/j.ympev.2009.03.021

Greenbaum E, Stanley EL, Kusamba C, Moninga WM, Goldberg SR, Bursey CR (2012) A new species of *Cordylus* (Squamata: Cordylidae) from the Marungu Plateau of south-eastern Democratic Republic of the Congo. African Journal of Herpetology 61(1): 14–39. https://doi.org/10.1080/21564574.2012.666505

Güçlü Ö, Candan K, Kankılıç T, Kumlutaş Y, Durmuş SH, Poulakakis N, Ilgaz Ç (2014) Phylogeny of *Trachylepis* sp. (Reptilia) from Turkey inferred from mtDNA sequences. Mitochondrial DNA 25(6): 456–463. https://doi.org/10.3109/19401736.2013.814108

Karin BR, Metallinou M, Weinell JL, Jackman TR, Bauer AM (2016) Resolving the higher-order phylogenetic relationships of the circumtropical *Mabuya* group (Squamata: Scincidae): An out-of-Asia diversification. Molecular Phylogenetics and Evolution 102: 220–232. https://doi.org/10.1016/j.ympev.2016.05.033

Lavin BR, Papenfuss TJ (2012) The phylogenetic position of *Chalcides ocellatus* (Squamata: Scincidae) from Yemen and Somalia. Zootaxa 3221(1): 26–36. https://doi.org/10.11646/zootaxa.3221.1.2

Noonan BP, Pramuk JB, Bezy RL, Sinclair EA, de Queiroz K, Sites Jr JW (2013) Phylogenetic relationships within the lizard clade Xantusiidae: Using trees and divergence times to address evolutionary questions at multiple levels. Molecular Phylogenetics and Evolution 69(1): 109–122. https://doi.org/10.1016/j.ympev.2013.05.017

Portik DM, Bauer AM, Jackman TR (2010) The phylogenetic affinities of *Trachylepis sulcata nigra* and the intraspecific evolution of coastal melanism in the western rock skink. African Zoology 45: 147–159. https://doi.org/10.3377/004.045.0217

Stanley EL, Bauer AM, Jackman TR, Branch WR, Mouton PLFN (2011) Between a rock and a hard polytomy: Rapid radiation in the rupicolous girdled lizards (Squamata: Cordylidae). Molecular Phylogenetics and Evolution 58(1): 53–70. https://doi.org/10.1016/j.ympev.2010.08.024

Stanley EL, Ceríaco LMP, Bandeira S, Valerio H, Bates MF, Branch WR (2016) A review of *Cordylus machadoi* (Squamata: Cordylidae) in southwestern Angola, with the description of a new species from the Pro-Namib desert. Zootaxa 4061(3): 201–226. https://doi.org/10.11646/zootaxa.4061.3.1

Townsend TM, Larson A, Louis E, Macey JR (2004) Molecular phylogenetics of Squamata: The position of snakes, amphisbaenians, and dibamids, and the root of the squamate tree. Systematic Biology 53(5): 735–757. https://doi.org/10.1080/10635150490522340

Weinell JL, Branch WR, Colston TJ, Jackman TR, Kuhn A, Conradie W, Bauer AM (2019) A species-level phylogeny of *Trachylepis* (Scincidae: Mabuyinae) provides insight into their reproductive mode evolution. Molecular Phylogenetics and Evolution 136: 183–195. https://doi.org/10.1016/j.ympev.2019.04.002

Whiting AS, Sites Jr JW, Pellegrino KCM, Rodrigues MT (2006) Comparing alignment methods for inferring the history of the New World lizard genus *Mabuya* (Squamata: Scincidae). Molecular Phylogenetics and Evolution 38(3): 719–730. https://doi.org/10.1016/j.ympev.2005.11.011
